# Ectomycorrhizal fungal diversity may be influenced by arbuscular mycorrhizal trees in mixed temperate forests

**DOI:** 10.1007/s00572-026-01267-2

**Published:** 2026-05-11

**Authors:** Andrew M. Cortese, Andrew C. Eagar, Sara M. Moledor, Kurt A. Smemo, Richard P. Phillips, Christopher B. Blackwood

**Affiliations:** 1 Department of Plant, Soil, and Microbial Sciences, Michigan State University, East Lansing, MI 48824 USA; 2 New York Natural Heritage Program, SUNY College of Environmental Science and Forestry, Albany, NY 12207 USA; 3 Department of Plant Biology, Michigan State University, East Lansing, MI 48824 USA; 4 Department of Environmental Science and Studies, Berry College, Mt. Berry, GA 30149 USA; 5 Environmental Studies and Science Department, Skidmore College, Saratoga Springs, NY 12866 USA; 6 Department of Biology, Indiana University, Bloomington, IN 47405 USA

**Keywords:** *Arbuscular mycorrhizae*, Biogeochemistry, *Ectomycorrhizae*, Non-host interactions, Temperate forests

## Abstract

Mycorrhizal fungi are critical plant mutualists that influence nutrient cycling, yet we have an incomplete understanding of factors driving their diversity and function. While it’s presumed that diverse host plant communities support diverse mycorrhizal fungal communities, abiotic factors and non-host plants may also be important. We investigated whether ectomycorrhizal fungal (ECMF) communities in soils and roots varied among plots differing in tree and soil properties in three mixed ectomycorrhizal (ECM)-arbuscular mycorrhizal (AM) forests in NY, USA. We used mixed-effects models and multivariate tests to investigate ECMF taxonomic and functional responses to (1) ECM tree dominance, (2) taxonomic and phylogenetic diversity of ECM and AM trees, and (3) soil properties. ECMF taxonomic diversity was most strongly related to ECM tree dominance and the diversity of AM – but not ECM – trees. ECMF functional diversity was mostly related to soil factors and to a lesser extent tree communities. In most cases, soil nitrogen negatively influenced ECMF diversity. Collectively, our results suggest non-host (i.e., AM) tree diversity is associated with increased ECMF diversity, potentially by modifying soil conditions and resource availability. As such, mixed plots dominated by trees associating with different mycorrhizal types may be hotspots for ECMF diversity, with consequences for ecosystem functioning.

**Supplementary Information:**

The online version contains supplementary material available at 10.1007/s00572-026-01267-2.

## Introduction

Mixed temperate forests in North America are often composed of a mosaic of arbuscular mycorrhizal (AM) and ectomycorrhizal (ECM) trees, representing the two globally dominant mycorrhizal types (Frelich et al. 1993; Eagar et al. 2020; Brundrett and Tedersoo 2020; Cortese et al. 2023). Under the current paradigm of plant-mycorrhizal interactions, communities of mycorrhizal fungi are driven predominantly by host plant vegetation (Ishida et al. 2007; Burrill et al. 2023), and non-hosts are generally not considered (Molina and Horton 2015). Therefore, the influence of non-host (e.g., AM) trees on ectomycorrhizal fungal (ECMF) communities remains largely unexplored, but they may play an important role in shaping ECMF in mixed AM-ECM forests. In addition to biotic interactions, abiotic properties also influence ECMF (Tedersoo et al. 2014b). It is well known that AM and ECM forest patches differentially affect soil biogeochemistry (Phillips et al. 2013), but it is unclear how these characteristics may indirectly influence ECMF communities. With further increases in the dominance of AM trees owing to global change (Averill et al. 2018; Jo et al. 2019), there is a pressing need to investigate how host and non-host trees affect ECMF communities in mixed temperate forests.

Ectomycorrhizal fungi (ECMF) are both taxonomically and functionally diverse (Tedersoo and Smith 2013; van der Heijden et al. 2015) and exhibit high species richness in temperate forests (Tedersoo et al. 2014b). In mixed AM-ECM forests, ECMF diversity can be positively related to ECM tree dominance, as plots dominated by ECM trees (in terms of total or relative ECM tree basal area) support the most diverse communities of ECMF (e.g., “ECM dominance hypothesis”; Spake et al. 2016; Eagar et al. 2023; Cortese et al. 2023). Additionally, ECMF community composition is influenced by ECM tree composition (Ishida et al. 2007; Twieg et al. 2007) owing to ECMF host specificity (Molina and Horton 2015). Thus, in many instances, positive relationships have been observed between diversity of ECMF and ECM plant species (Bills et al. 1986; Peay et al. 2013; Tedersoo et al. 2016; Otsing et al. 2021) with some reports of stronger relationships with ECM tree phylogenetic diversity (Tedersoo et al. 2013; Nguyen et al. 2016b; Miyamoto et al. 2022). However, it remains unclear whether the dominance, taxonomic diversity, or phylogenetic diversity of ECM trees best predict ECMF diversity in mixed AM-ECM forests.

In mixed AM-ECM forests, forest patches dominated by AM trees generally exhibit higher litter decomposition rates, soil pH, inorganic nutrients, and abundance of soil saprotrophs relative to patches dominated by ECM trees (Melillo et al. 1982; Read 1991; Hobbie 1992; Cornelissen et al. 2001; Lovett and Mitchell 2004; Phillips and Fahey 2006; Eagar et al. 2022). AM trees usually exhibit greater fine root proliferation than ECM trees, likely reflecting nutrient-acquisitive growth strategies (Chen et al. 2016; Valverde-Barrantes et al. 2018). Interactions between intertwined AM-ECM root systems may therefore result in competition for resources which may decrease ECM root density and influence fungal composition (McHugh and Gehring 2006; Peay et al. 2011; Fernández et al. 2022; Barou et al. 2023; Xie et al. 2024). ECMF may further respond to AM community spillover effects if soil nutrient cycling and microbial communities shift to those more characteristic of the surrounding AM vegetation (Phillips et al. 2013; Cheeke et al. 2017; Eagar et al. 2022, 2024). Moreover, spillover effects may result in unpredictable soil conditions, as mixtures of functionally distinct plant litter (e.g., AM and ECM) can exhibit emergent turnover characteristics (Cornelissen et al. 2001; Jacobs et al. 2018; Grossman et al. 2020) that diverge from “typical” AM-ECM nutrient economies (Phillips et al. 2013; Midgley and Phillips 2014).

In addition to drivers of ECMF taxonomic diversity, controls on the range of ECMF functional traits present in a community are also important to understand. ECMF are important regulators of terrestrial carbon (C) cycling (Averill et al. 2014; Averill and Hawkes 2016; Fernandez and Kennedy 2016; Fernandez et al. 2016) and exhibit differential capabilities to enzymatically liberate nutrients from complex organic sources (Bending and Read 1995; Hobbie and Agerer 2010; Op De Beeck et al. 2018; Pellitier and Zak 2018; Okada et al. 2024). Classification of ECMF taxa into exploration types based on the extent and hydrophobicity of their extramatrical mycelium has served as a useful approach to characterize ECMF functional traits, which may correspond with their ability to explore and break down organic substrates (Agerer 2001; Hobbie and Agerer 2010; but see Jörgensen et al. 2023). However, production of extensive mycelium requires high inputs of C by host trees (Cairney 2012), which is governed by soil nutrient availability (Högberg et al. 2003, 2021; Phillips et al. 2011; Corrêa et al. 2011). For example, medium distance-fringe ECMF produce extensive extramatrical mycelium with high enzymatic capacity to extract nitrogen (N) from recalcitrant leaf litter (Hobbie and Agerer 2010; Lilleskov et al. 2011; Bödeker et al. 2014); yet, under high soil N availability, supporting ECMF with this capability may be less cost-efficient than other ECMF for host trees (Kiers et al. 2011; Bunn et al. 2024). Therefore, elevated soil N from inputs of labile AM leaf litter may select for ECMF that produce less extramatrical mycelium, such as those forming the contact exploration type (Arnolds 1991; Avis et al. 2003; Lilleskov et al. 2011; Koide et al. 2014; Looney et al. 2018).

In our study, we test competing hypotheses comparing the importance of both ECM and AM tree dominance, taxonomic diversity, and phylogenetic diversity on ECMF taxonomic and functional (exploration type) diversity. We also investigate relationships between ECMF diversity and soil conditions consistent with an ECM nutrient economy (e.g., low inorganic N, high C: N ratio, and low soil pH). We hypothesize that (H1) ECMF diversity and composition are primarily influenced by ECM tree dominance and diversity and may be secondarily influenced by non-host (AM) trees, total tree abundance, or soil properties and (H2) ECMF functional group diversity and composition will be more strongly affected by soil conditions than ECMF taxonomic diversity and composition. To test these hypotheses, here we build on analyses in a previously published study (Eagar et al. 2023) in which we found that community dominance by AM versus ECM trees broadly influenced fungal community composition and abundance across fungal functional groups. However, Eagar et al. (2023) did not address the potential effects of tree diversity or non-host trees on ECMF composition and diversity. The more detailed analysis of ECMF we provide below is warranted because groups of potential host and non-host trees can be delineated (Brundrett and Tedersoo 2020; Soudzilovskaia et al. 2020), unlike with most other fungal functional groups.

## Methods

### Study design

For our study, we re-analyzed data from Eagar et al. (2023) which investigated the effects of focal tree mycorrhizal associations (AM versus ECM), relative ECM basal area of the surrounding tree communities, and site and soil characteristics on fungal community composition in the Adirondack Mountains of New York, USA. Tree community composition, soil biogeochemical characteristics, and fungal communities from soil and tree root samples were measured from 72 plots distributed evenly across three sites (24 each at Shingle Shanty Preserve [43.894, -74.732], Huntington Wildlife Forest [43.987, -74.245], and Lake George Wild Forest [43.661, -73.545]). At each site, 24 plots (15 m radius) were established around a dominant focal AM or ECM tree for a total of 12 plots per focal tree mycorrhizal type. Basal area of all trees > 2 cm diameter at breast height (DBH; 1.37 m) were measured. The 72 sampled plots represent a gradient of 0.0–77.8% AM tree basal area (100.0–22.2% ECM tree basal area). Thus, our study contains plots with a prominent, focal AM or ECM tree surrounded by a tree community that varies in its mycorrhizal composition. The full study design and sampling methods can be found in the main text and supplementary information for Eagar et al. (2023).

#### Tree community and soil characteristics

For each tree species, mycorrhizal types were assigned following Brundrett and Tedersoo (2020) and checked in the FungalRoot database (Soudzilovskaia et al. 2020; see Table S1 for tree mycorrhizal assignments and abundances at each site). While there are a few reports of some ECM tree species included in our study associating with AM fungi (Vozzo and Hacskaylo 1974; Dickie et al. 2001; Teste et al., 2020; Fahey et al. 2025), AM associations of these trees likely occur transiently prior to ECM colonization (Lapeyrie and Chilvers 1985) and are thus categorized as ECM (Brundrett and Tedersoo 2020; Soudzilovskaia et al. 2020). *Populus grandidentata*, which readily forms AM and ECM (Brundrett and Tedersoo 2020), was included as an ECM host tree but was only present in Deer Leap at low abundance.

Briefly, we calculated various measures of taxonomic and phylogenetic diversity for the AM, ECM, and combined AM and ECM tree communities in each plot. We calculated both Hill ^*0*^*D* (species richness) and Hill ^*2*^*D* (inverse Simpson’s index; Chao et al. 2014) for all tree communities (Table S2). We also calculated plot-level pairwise Sørensen phylogenetic dissimilarity for AM, ECM, and combined AM and ECM tree communities using the packages *adviv* (Pavoine 2020) and *betapart* (Baselga and Orme 2012) and converted them into phylogenetic eigenvectors using principal coordinate analysis (PCoA) with the package *vegan* (Oksanen 2019). The full procedures for calculation of taxonomic and phylogenetic tree diversity can be found in the Supporting Information (Supporting Methods 1) as well as variable summaries (Table S2). We also calculated the following measures of ECM dominance: focal tree mycorrhizal type (ECM or AM), ECM tree basal area and stem density, relative ECM tree basal area and stem density, and an ECM tree importance value (Whittaker 1965) using summed relative ECM tree basal area and stem density (Table S3).

A suite of soil variables were measured to characterize the soil conditions at each plot (see Eagar et al. 2023 for the full procedure). Briefly, the upper forest floor (O_i_ and O_e_ horizons) mass was measured, composite 5 cm wide × 10 cm deep soil cores (Oa-mineral soil) were collected to assess fine root biomass, total C, total N, and C: N ratio, and inorganic N, and soil respiration was also measured in each plot (Table S4).

#### Molecular identification of ECM fungi and bionformatics

The full procedures for molecular identification of ECM fungi and bioinformatics can be found in Eagar et al. (2023). Briefly, composite soil and root samples for molecular analysis were collected from 3 locations within 3 m of the base of focal trees in each plot using a 2.5 cm wide × 15 cm deep soil probe. Roots were sorted out from each sample by passing soils through a 2 mm sieve. DNA was extracted from roots using the CTAB method (Gardes and Bruns 1993) and from soil using Qiagen DNeasy PowerSoil^®^ kits (Qiagen^®^, Hilden, Germany).

We amplified the internal transcribed spacer (ITS2) region using triplicate PCR reactions with the forward primers ITS3ngs1-3 and ITS3ngs4-5 paired with the reverse primer ITS4ngsUni (Tedersoo and Lindahl 2016). For each sample, PCR product was pooled, barcoded, and submitted for 2 × 300 bp Illumina^®^ MiSeq sequencing at the Ohio State University Molecular and Cellular Imaging Center (Wooster, OH, USA).

Demultiplexed sequences were processed via QIIME2 v.2019.7 (Bolyen et al. 2019). Primers were removed with cutadapt (Martin 2011) prior to quality filtering and amplicon sequence variant (ASV) clustering in DADA2 (Callahan et al. 2016). ASVs were assigned taxonomy from the UNITE database ver. November 18, 2018 (UNITE Community, 2019) via a naïve Bayesian classifier (Bokulich et al. 2018). Fungal functional roles were assigned using FUNGuild ver. 1.1 (Nguyen et al. 2016a). Abundances of the ECMF genera can be found in the Supporting Information (Table S6; Figs. S2-S5). We grouped ECMF into respective exploration types (contact, long distance, mat, medium distance fringe, medium distance smooth, short distance coarse, short distance delicate, and unknown; Agerer 2001) using the FungalTraits database (Põlme et al. 2020) and omitted all “unknown” exploration types from our functional group analysis (*Hydnobolites*; 0.11% of sequence reads). We then estimated the abundance of medium distance-fringe ECMF by calculating the proportion of sequence reads out of the total number of reads (excluding “unknown” exploration types) per sample. We chose to investigate this group because they are low-nutrient specialist ECMF that are sensitive to nitrogen (Lilleskov et al. 2011).

### Statistical analyses

*Correlations of tree and soil variables.* We conducted all analyses in the RStudio statistical environment using R version 4.2.2 (R Core Team, 2022). We tested for Pearson correlations (*α* = 0.05) between measured tree diversity, tree abundance, and soil variables to investigate relationships between soils and tree composition consistent with either AM or ECM nutrient economies (Phillips et al. 2013).

*Modeling ECMF taxonomic diversity.* We used a multi-step model selection procedure to model the effects of tree diversity, tree abundance, and soil conditions on ECMF taxonomic Hill ^*0*^*D* (i.e., genus richness) and Hill ^*2*^*D* (i.e., inverse Simpson index) from soils and roots. The model selection procedure was designed to remove uninformative variables and select the most parsimonious models (Burnham & Anderson,z 2004). We then used tree abundance and soil variables included in the first modeling procedure as covariates to compare the influence of each measure of tree diversity on ECMF taxonomic Hill ^*0*^*D* and Hill ^*2*^*D*. The full diversity modeling procedure can be found in the Supporting Information (Supporting Methods 2).

Briefly, we used linear mixed-effects regressions, with site as a random effect, with the package *lme4* (Bates et al. 2014). We constructed separate global models containing each suite of AM, ECM and combined AM-ECM tree diversity variables (Table S2), AM, ECM, and combined AM-ECM tree abundance (Table S3), and soil variables (Table S4) to identify the most informative variables from each suite. Variance inflation factor (VIF) was used to reduce multicollinearity (Borcard et al. 2018). We then conducted *unsupervised* variable selection using corrected Akaike’s Information Criterion (AICc) for each global model containing each variable suite with the dredge() function with the package *MuMIn* (Bartoń 2023). We selected the variables included in each top-ranked model for *supervised* model selection in which we fit separate linear mixed-effects models containing variables individually, or in biologically relevant combinations with a maximum of one tree abundance and one diversity variable in each model, in addition to any soil variables. We then ranked all candidate models with AICc using the package *AICcmodavg* (Mazerolle 2023). To account for uncertainty due to AICc-based model selection, we model-averaged ECMF diversity responses to predictors included in all top models based off of model weight (Burnham & Anderson, 2004) using the package *AICcmodavg*. We then determined predictor variables to be significant if their 95% confidence intervals did not intersect zero.

*Identifying tree diversity variables that best predict ECMF taxonomic diversity.* We conducted an additional modeling step to verify the importance of tree diversity variables identified in the previous modeling step. We directly compared the influence of all measured AM, ECM, and AM-ECM tree variables on ECMF taxonomic diversity by specifying a global model containing all measured tree diversity variables (Table S2) as well as any overall tree abundance and soil effects variables that were included in the most parsimonious model above (Table S7). After omitting any variables with VIF ≥ 10, we then used the dredge() function to rank all models from each model set with AICc, and then model-averaged all tree diversity responses with *MuMIn*.

*Comparing tree and ECMF community composition.* To investigate direct relationships between communities of trees and ECMF, we used Mantel tests to compare ECMF taxonomic composition with AM, ECM, and combined AM-ECM tree taxonomic and phylogenetic composition. We used Bray-Curtis distance for taxonomic composition of ECMF and AM, ECM, and combined AM-ECM tree communities and Sørenson distance for pairwise phylogenetic dissimilarity of AM, ECM, and combined AM-ECM tree communities.

We then explored how ECMF taxonomic composition is influenced by AM, ECM, and combined AM-ECM taxonomic and phylogenetic diversity and composition, ECM dominance and other measures of tree abundance, and soil characteristics. We used forward step selection of partial redundancy analysis (RDA) models using the function ordiR2step() in *vegan* (Oksanen et al. 2013) with Hellinger-transformed ECMF community (Borcard et al. 2018) and site as a conditioning variable. The full list of included variables (except for AM, ECM, and combined AM-ECM phylogenetic eigenvectors) can be found in the Supporting Information (Tables S2-S4). We conducted individual forward selections for each suite of variables similar to the approach outlined for modeling diversity responses and used the vif.cca() function to remove any collinear variables (VIF ≥ 10). We then used the goodness() command to identify ECMF taxa that had high variation explained (R^2^ *≥* 0.10) by RDA axes. Finally, we tested Pearson correlations between AM tree species and the AM tree phylogenetic eigenvector and with soil pH to further explore how individual AM tree species influenced ECMF taxonomic composition and relate to soil conditions.

*ECMF functional groups and relative abundance of medium distance-fringe exploration type*. To model ECMF functional diversity, we used exploration type assignments from the FungalTraits database (Agerer 2001; Põlme et al. 2020) as a proxy for functional groups and then followed the same modeling procedures as previously described for ECMF taxonomic diversity. We also modeled the composition of ECMF functional groups using the same multivariate procedures (Mantel tests and partial RDA model selection) as described for ECMF taxonomic composition. We also investigated the influence of tree composition and soils on the relative abundance of medium distance-fringe ECMF. We followed a model selection procedure similar to the one previously described for ECMF taxonomic and functional diversity, except we used zero-inflated beta regressions with the package *glmmTMB* (Brooks et al. 2017) to account for overdispersion.

## Results

### Relationship between tree and soil variables

Although we did not detect strong AM-ECM nutrient effects between AM and ECM dominated patches, we found several correlations between tree composition and soil conditions (Table S5). Most notably, we found that soil pH was negatively correlated and C: N ratio was positively correlated with percent ECM tree basal area (*r* = -0.25 and *r* = 0.31, respectively), while other soil characteristics were largely independent of ECM dominance (Table S5). Interestingly, we found that AM, ECM, and combined AM-ECM tree Hill ^*0*^*D* were each positively correlated with soil pH (*r* = 0.40, *r* = 0.45, and *r* = 0.52, respectively). Additionally, AM tree phylo-Hill ^*0*^*D* was negatively correlated with N mineralization rate (*r* = -0.25), while AM tree phylo-Hill ^*0*^*D* and phylo-Hill ^*2*^*D* were both negatively correlated with soil N (*r* = -0.39 and *r* = -0.37, respectively) and carbon (*r* = -0.37 and *r* = -0.35; Table S5).

### ECMF taxonomic diversity

**Fig. 1:**
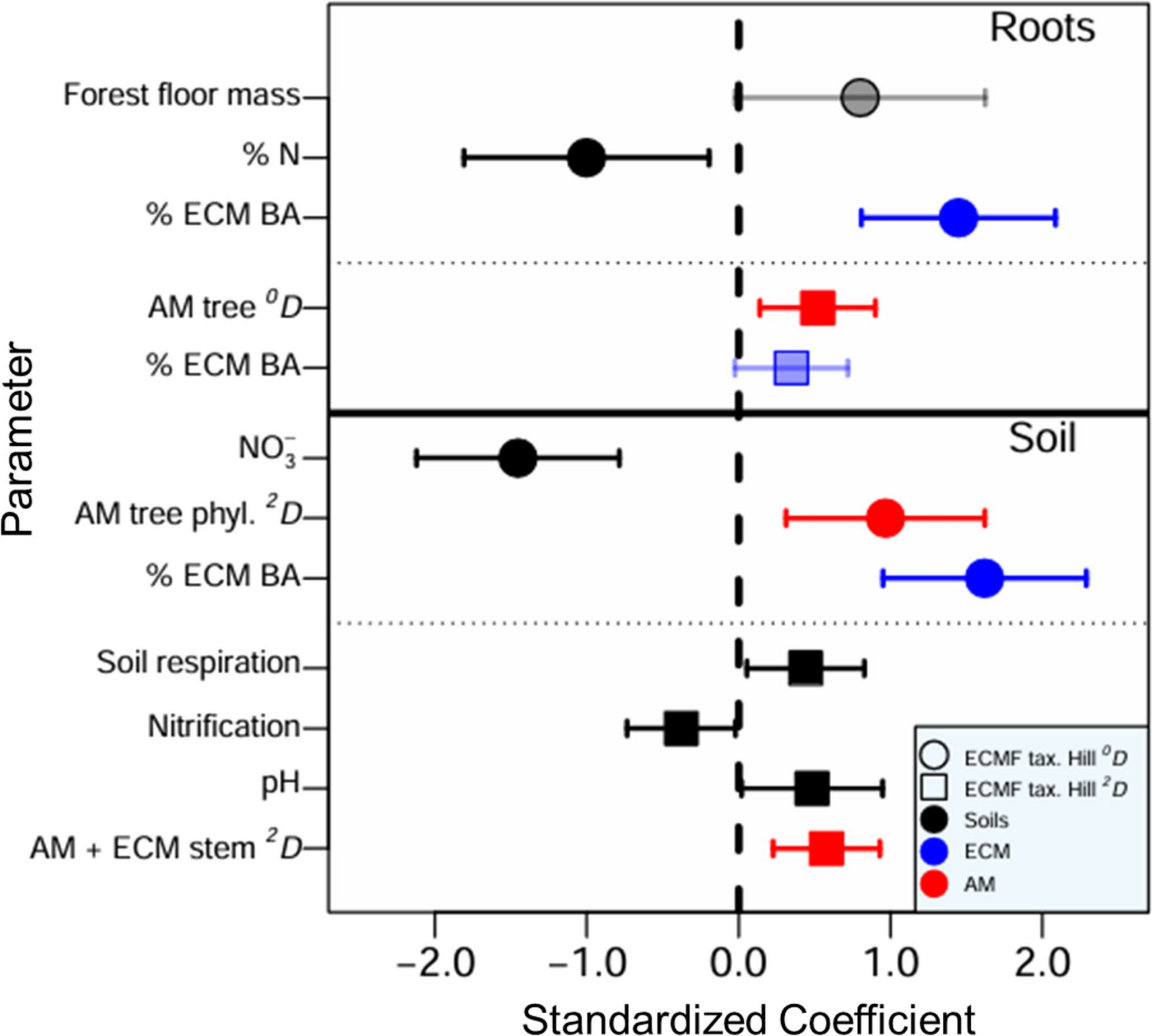
Model averaged responses of ECMF taxonomic Hill ^*0*^*D* (circles) and Hill ^*2*^*D* (squares) from roots (top) and soil (bottom) to tree composition and soil variables included in top AICc-ranked models (Table S7) from the Adirondack Mountains, USA (2017). Bolded points indicate significant responses with 95% confidence intervals that do not intersect zero (dashed line). Red symbols are explanatory factors that include AM tree characteristics (and may also include ECM tree characteristics), whereas blue symbols include only ECM tree characteristics. BA = basal area and phyl. = phylogenetic

**Fig. 2:**
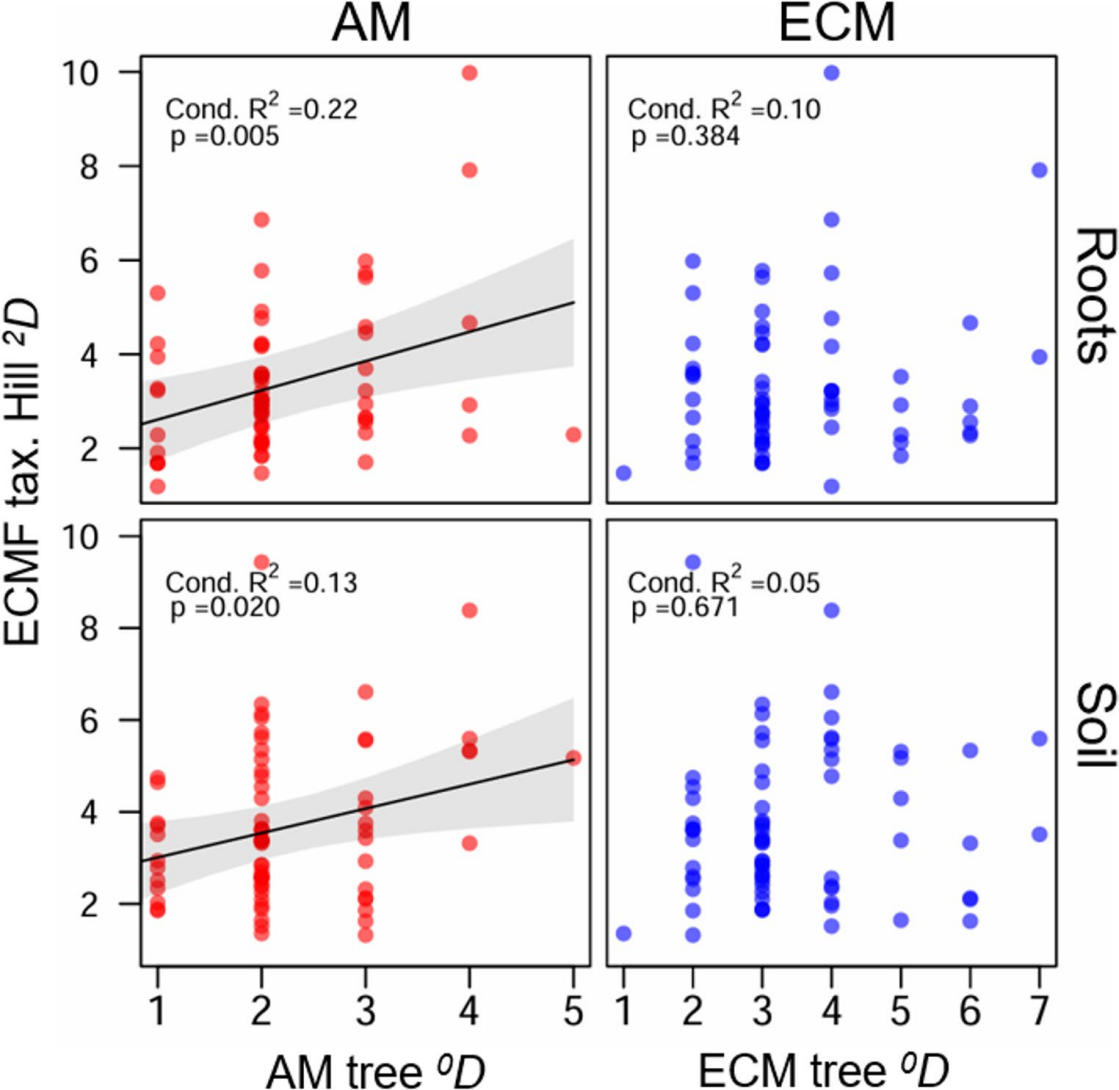
ECMF taxonomic Hill ^*2*^*D* from roots (top) and soil (bottom) to measures of AM (left; red points) and ECM (right; blue points) tree Hill ^*0*^*D* (species richness) from the Adirondack Mountains, USA (2017). Regression lines and 95% confidence intervals are only shown for significant (*p* < 0.05) relationships

In three out of four cases, the top-ranked models for ECMF taxonomic diversity included significant, positive effects of non-host (AM) trees, while no top models included the effect of ECM tree diversity alone (Table S7; Fig. 1). The measures of ECMF diversity significantly influenced by AM tree diversity included soil ECMF-Hill ^*0*^*D* and ECMF-Hill ^*2*^*D*, and root ECMF-Hill ^*2*^*D*. Top models explained 26–51% of the variation in ECMF diversity (conditional R^2^, Table S7). These patterns were also confirmed when no other predictors were included in the model, where we observed significant, positive relationships between root and soil ECMF Hill ^*2*^*D* and AM tree Hill ^*0*^*D*, but not ECM tree Hill ^*0*^*D* (Fig. 2).

We also found significant, positive effects of ECM tree dominance (percent ECM basal area) on soil and root ECMF-Hill ^*0*^*D*, as well as a nonsignificant, positive effect on root ECMF-Hill ^*2*^*D*. Among soil variables, three out of four top-ranked models showed significant, negative effects of soil N on ECMF taxonomic diversity (soil ECMF-Hill ^*0*^*D*, soil ECMF-Hill ^*2*^*D*, and root ECMF-Hill ^*0*^*D*). The top-ranked models also included significant, positive effects of soil respiration and soil pH on soil ECMF-Hill ^*2*^*D* as well as a non-significant, positive effect of forest floor mass on root ECMF-Hill ^*0*^*D* (Table S7; Fig. 1).

Models testing all tree diversity variables, while accounting for the tree dominance and soil variables selected above, confirmed results described above. The only additional tree diversity variable found to have a significant effect on ECMF diversity was a weak positive effect of ECM tree basal area Hill ^*2*^*D* on soil ECMF taxonomic Hill ^*0*^*D* (Fig. S6; Tables S7-S8).

### ECMF community composition

Among all comparisons between tree and ECMF genus composition, stronger relationships were found between pairwise phylogenetic distance compared to taxonomic distance for AM, ECM, and combined AM-ECM tree communities (Table 1). Between measures of phylogenetic distance, combined AM-ECM trees explained slightly more variation for soil (Mantel *r* = 0.233) and roots (Mantel *r* = 0.293) than ECM trees alone for soil (Mantel *r* = 0.223) and roots (Mantel *r* = 0.271). However, AM trees alone did not explain any variation in.

ECMF genus composition for soil (Mantel *r* = -0.024) or roots (Mantel *r* = -0.039; Table 1).

**Table 1:** Mantel test results comparing composition of ECMF genera from soils (top) and roots (bottom) with different measures of taxonomic and phylogenetic composition of AM, ECM, as well as AM-ECM tree communities in the Adirondack Mountains, USA (2017)

Substrate	Tree composition	Mantel *r*	*p*
Soil	Combined AM-ECM trees	0.092	0.073
	ECM trees only	0.175	0.005
	AM trees only	-0.024	0.631
	Combined AM-ECM tree phylogenetic distance	0.233	0.002
	ECM tree phylogenetic distance only	0.223	0.004
	AM tree phylogenetic distance only	0.170	0.003
Roots	Combined AM-ECM trees	0.131	0.019
	ECM trees only	0.227	0.002
	AM trees only	-0.039	0.709
	Combined AM-ECM tree phylogenetic distance	0.293	0.001
	ECM tree phylogenetic distance only	0.271	0.001
	AM tree phylogenetic distance only	0.240	0.002

**Fig. 3:**
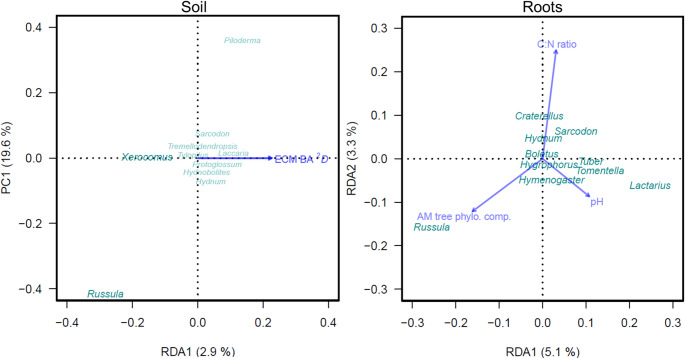
Partial redundancy analysis (RDA) biplots showing composition of the top ten ECMF genera with the most variation explained by RDA axes from soils (left; *N* = 66) and roots (right; *N* = 60) sampled from the Adirondack Mountains, USA (2017). Vector arrows represent top variables identified from stepwise RDA model selection; bolded text indicates genera with *r* ≥ 0.10 to RDA axes. BA = basal area and phylo. comp. = phylogenetic composition

Stepwise RDA model selection found a significant relationship between soil ECMF composition and ECM tree basal area Hill ^*2*^*D* (*p* = 0.023; R^2^ = 0.03). Among genera detected in soil, *Russula* and *Xerocomus* had the most variation explained (R^2^ = 0.10 and R^2^ = 0.11, respectively) by the RDA axis (Fig. 3). We also found significant relationships between root ECMF composition and AM tree pairwise phylogenetic composition, as well as soil C: N ratio and pH (*p* < 0.001; R^2^ = 0.10). Among genera detected in roots, *Boletus* had the most variation explained by RDA axes (R^2^ = 0.27), followed by *Tuber* (R^2^ = 0.19), *Craterellus* (R^2^ = 0.17), *Hymenogaster* (R^2^ = 0.16), *Lactarius* (R^2^ = 0.16), and then *Russula*, (R^2^ = 0.15; Fig. 3).

### ECMF functional diversity

**Fig. 4:**
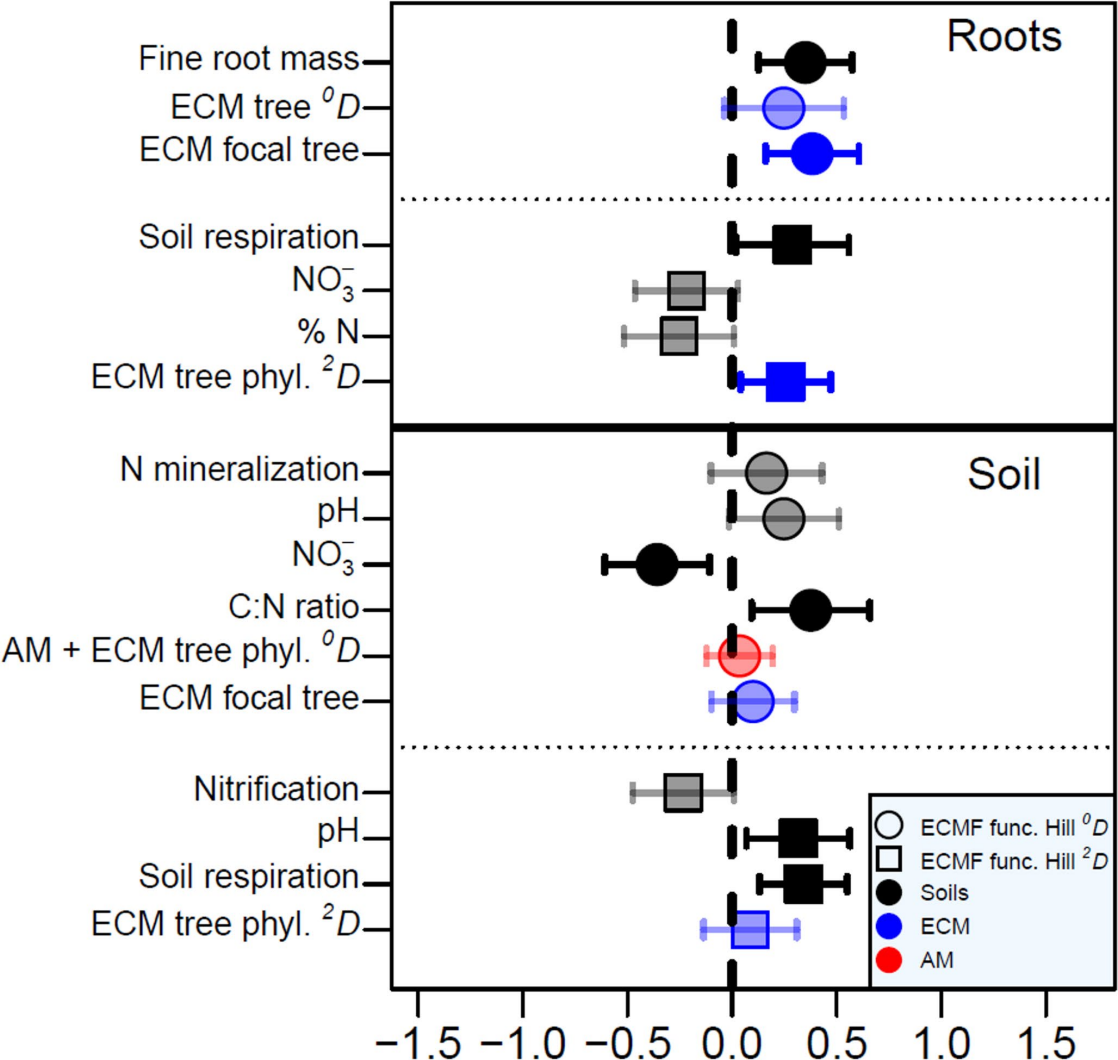
Model averaged responses of ECMF functional Hill ^*0*^*D* (circles) and Hill ^*2*^*D* (squares) from roots (top) and soil (bottom) to tree composition and soil variable s included in top AICc-ranked models (Table S8) from the Adirondack Mountains, USA (2017). Bolded points indicate significant responses with 95% confidence intervals that do not intersect zero (dashed line). Red symbols are explanatory factors that include AM tree characteristics (and may also include ECM tree characteristics), whereas blue symbols include only ECM tree characteristics (see legend in Fig. 1). Phyl. = phylogenetic

In contrast to ECMF taxonomic diversity, ECMF functional diversity was primarily influenced by soil conditions and to a lesser degree by ECM trees, but not significantly by AM trees (Fig. 4). We found that ECM tree diversity had a significant, positive effect on root ECMF func-Hill ^*2*^*D*. The combined AM-ECM tree phylogenetic Hill ^*0*^*D*, which was the only variable containing AM trees in top-ranked models, had a non-significant, positive effect on soil ECMF func-Hill ^*0*^*D* (Table S8; Fig.4). We also found that ECM focal trees had a significant, positive effect on ECMF func-Hill ^*0*^*D* from roots but was non-significant from soil. Among soil variables, we found significant, negative effects of soil NO_3_^−^ and significant, positive effects of C: N ratio on ECMF func-Hill ^*0*^*D* from soil. Soil respiration and pH both had significant, positive effects on ECMF func-Hill ^*2*^*D* from soil, while soil respiration alone had a significant, positive effect from roots. Finally, fine root biomass had a significant, positive effect on root ECMF func-Hill ^*0*^*D* (Table S8; Fig. 4).

Models testing all tree diversity variables, while accounting for the tree dominance and soil variables selected above, confirmed results described above. Additionally, ECM tree basal area Hill ^*2*^*D*, which was omitted from our model selection procedure due to multicollinearity, had a significant, positive effect on soil ECMF func-Hill ^*0*^*D* (Fig. S7).

### ECMF functional composition

Among all comparisons between tree and ECMF functional group composition, stronger relationships were found between the pairwise phylogenetic distance compared to taxonomic distance for AM, ECM, and combined AM-ECM tree communities (Table 1). Between measures of phylogenetic distance, combined AM-ECM trees explained slightly more variation for soil (Mantel *r* = 0.147) and roots (Mantel *r* = 0.269) than ECM trees alone for soil (Mantel *r* = 0.140) roots (Mantel *r* = 0.257; Table 2).

**Table 2:** Mantel test results comparing composition of ECM functional groups from soils (top) and roots (bottom) with different measures of taxonomic and phylogenetic composition of AM, ECM, as well as combined AM-ECM tree communities in the Adirondack Mountains, USA (2017)

Substrate	Tree composition	Mantel *r*	*p*
Soil	Combined AM-ECM trees	0.076	0.088
	ECM trees only	0.113	0.031
	AM trees only	0.008	0.411
	Combined AM-ECM tree phylogenetic distance	0.147	0.009
	ECM tree phylogenetic distance only	0.140	0.008
	AM tree phylogenetic distance only	0.102	0.040
Roots	Combined AM-ECM trees	0.137	0.004
	ECM trees only	0.179	0.003
	AM trees only	0.029	0.286
	Combined AM-ECM tree phylogenetic distance	0.269	0.001
	ECM tree phylogenetic distance only	0.257	0.001
	AM tree phylogenetic distance only	0.240	0.001

**Fig. 5:**
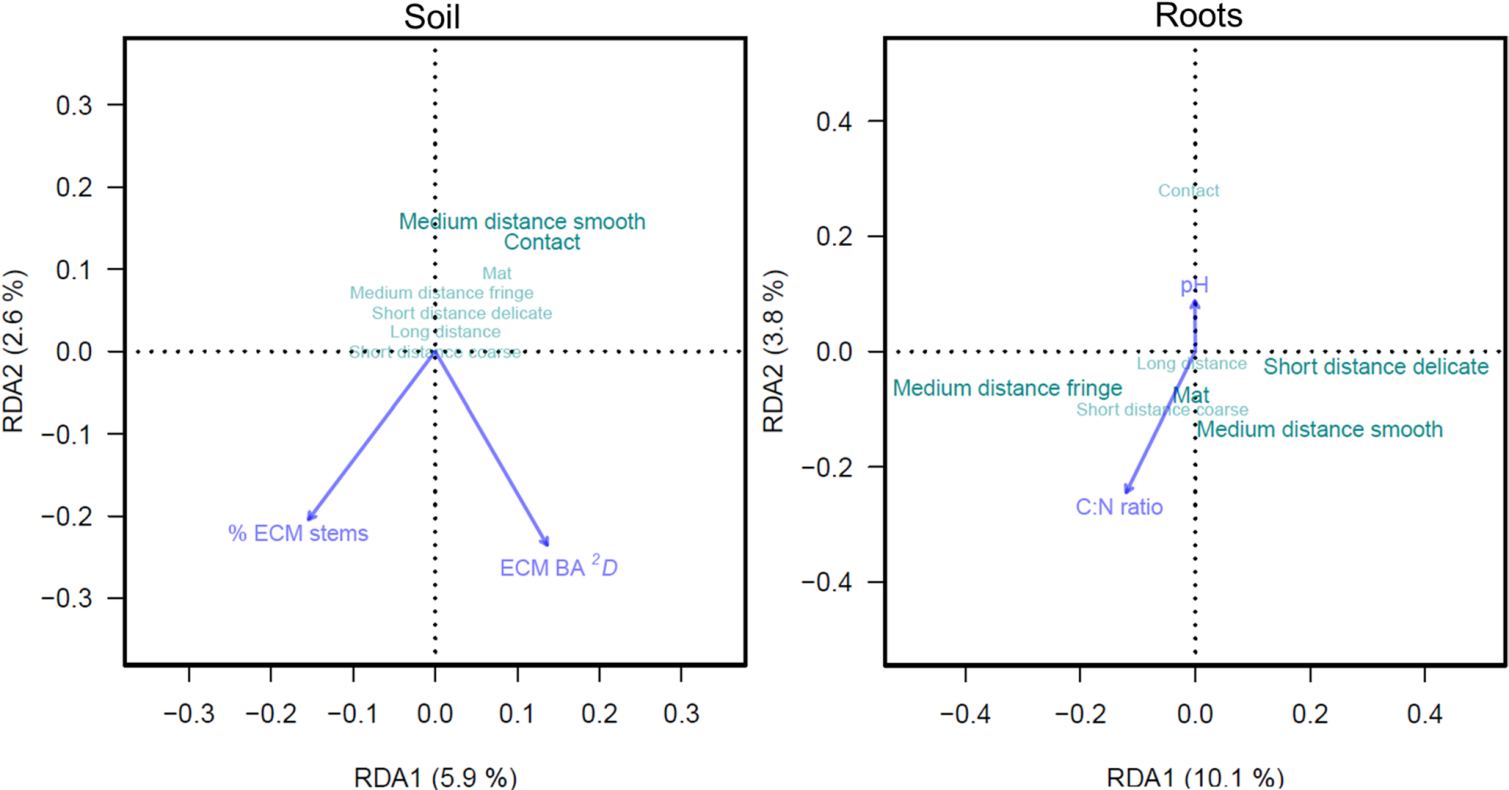
Partial redundancy analysis (RDA) biplots showing ECMF functional groups (Agerer 2001) with the most variation explained by RDA axes from soils (left; *n* = 66) and roots (right; *n* = 60) sampled from the Adirondack Mountains, USA (2017). Vector arrows represent top variables identified from RDA model selection and bolded text indicates functional groups with R^2^ ≥ 0.10

Stepwise RDA model selection found a significant relationship between the composition of ECMF functional groups from soil and the % of ECM tree stems as well as ECM tree basal area Hill ^*2*^*D* (*p* = 0.008; R^2^ = 0.08). Among functional groups, medium distance-smooth (R^2^ = 0.15) and contact (R^2^ = 0.13) exploration types have the most variation explained by the RDA axes (Fig. 5). We found a significant relationship between the ECMF functional composition from roots with soil pH and C: N ratio (*p* = 0.001; R^2^ = 0.14). Among groups, medium distance-fringe (R^2^ = 0.16), short distance-delicate (R^2^ = 0.12), medium distance-smooth (R^2^ = 0.12), and mat (R^2^ = 0.11) exploration types had the most variation explained by the RDA axes (Fig. 5).

### Relative abundance of medium distance-fringe exploration type

**Fig. 6:**
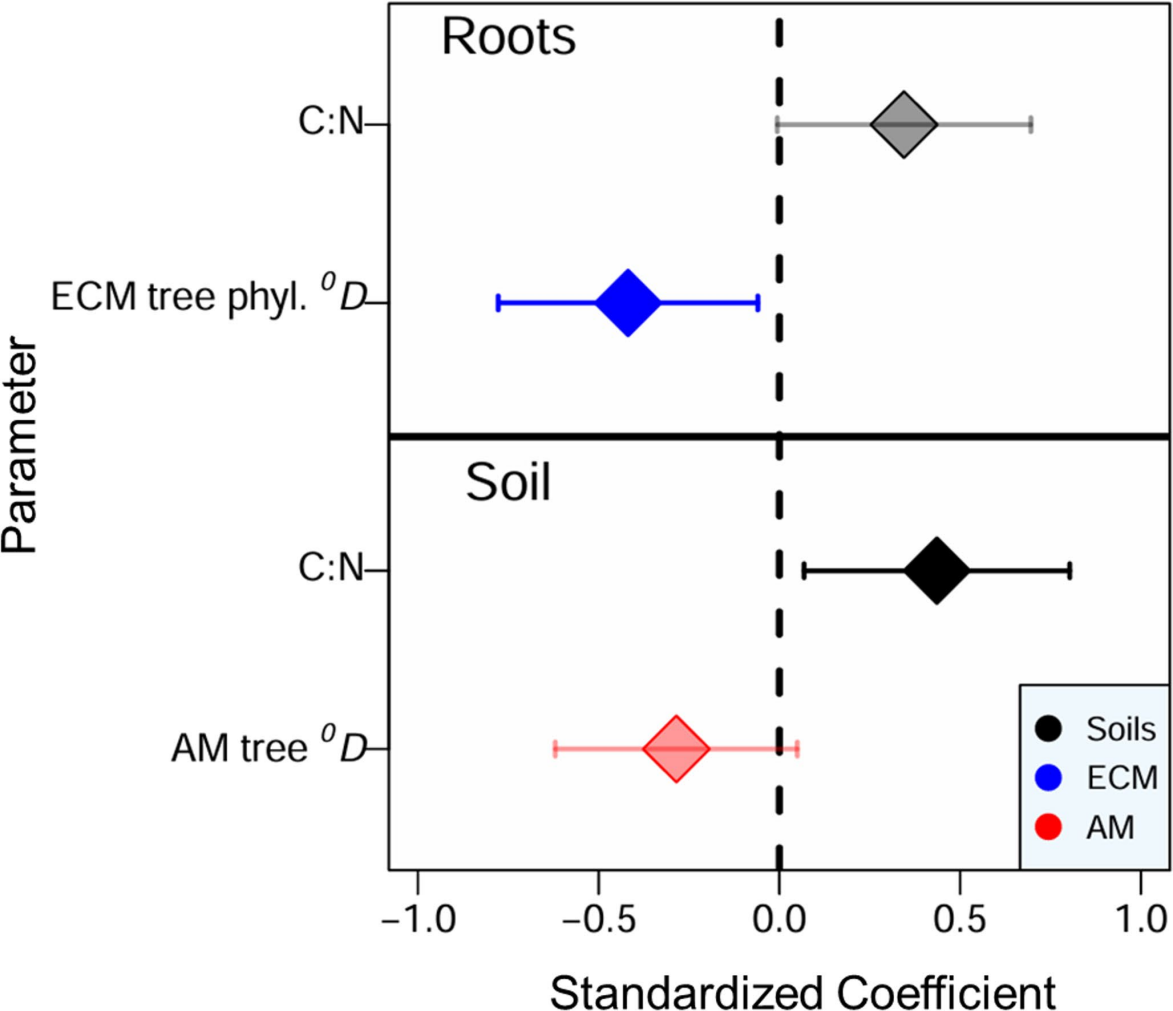
Model averaged responses showing relative abundance of medium distance-fringe ECMF from roots (top) and soil (bottom) in relation to tree diversity and soil variables included in top AICc-ranked model (Table S9) from the Adirondack Mountains, USA (2017). Bolded points indicate significant responses with 95% confidence intervals that do not intersect zero (dashed line)

We found conflicting results for the relative abundance of the medium distance-fringe exploration type ECMF between soil and roots. The top-ranked model for the relative abundance of medium distance-fringe ECMF from soil included a significant, positive effect of soil C: N ratio as well as a non-significant, negative effect of AM tree Hill ^*0*^*D*. In contrast, the top model from roots included a significant, negative effect of ECM tree phylogenetic Hill ^*0*^*D* as well as a non-significant, positive effect of soil C: N ratio (Table S9; Fig. 6).

## Discussion

In our study, we explored ECMF taxonomic and functional responses to AM, ECM, and combined AM-ECM tree diversity, mycorrhizal dominance, and soil characteristics from mixed AM-ECM temperate forests. Although we had previously shown that ECMF taxonomic diversity was related to ECM tree dominance (Eagar et al. 2023), here we found that the relationships between diversity and composition of ECMF and overstory trees were often stronger when including the AM tree community, as compared to the ECM tree community alone. In agreement with our first hypothesis (H1), ECM tree diversity was positively related to ECMF taxonomic and functional diversity and composition. However, contrary to our expectations, we found even stronger positive diversity relationships between AM trees and ECMF and also found evidence that AM trees influence ECMF composition. Collectively, our results suggest that while ECM host trees are important, non-host plant-fungal interactions also influence ECMF composition in mixed AM-ECM temperate forests. Among soil characteristics, we found overall negative effects of soil N on ECMF taxonomic diversity. In agreement with our second hypothesis (H2), we found that ECMF functional diversity and composition was primarily influenced by soil characteristics like C: N ratio and pH, but we also detected some functional responses to ECM dominance and tree diversity.

Our results offer support for the ECM dominance hypothesis (Spake et al. 2016; Eagar et al. 2023; Cortese et al. 2023) and suggest that ECM-dominated patches composed of high overall tree species diversity (including AM species) are hotspots of ECMF diversity in mixed AM-ECM temperate forests. Although tree composition was largely independent of AM-ECM nutrient syndromes in our study, we found that some soil conditions consistent with an ECM nutrient economy (e.g., low pH and high C: N ratio; Phillips et al. 2013) corresponded with greater ECMF dominance. In contrast to ECMF taxonomic diversity, functional diversity was generally less sensitive to tree composition and was largely influenced by soil conditions. Among exploration types, low-N specialist medium distance-fringe ECMF (Lilleskov et al. 2011) were more abundant in sites with high C: N ratio and, interestingly, were negatively related to ECM tree diversity.

### Non-host effects on ECMF in mixed AM-ECM forests

The positive associations of AM tree diversity and ECMF that we observed here are likely conditional on the presence of some ECM trees. Forest patches completely dominated by AM trees can exhibit comparatively low ECMF sequence diversity in soils (Eagar et al. 2022) and exceedingly low ECMF colonization of establishing ECM seedlings (Cortese and Horton 2023, 2024). ECMF generally do not form functional mycorrhizas with roots of AM trees (Brundrett 2009; Smith and Read 2010), yet ECM colonization of AM trees has been observed (Fruleux et al. 2023). While the mechanisms are unclear, the abundance of some ECMF taxa can be enhanced in mixtures of AM and ECM trees, likely due to an array of complicated belowground interactions (Heklau et al. 2021).

Among measures of tree composition, AM tree phylogenetic composition had the strongest association with ECMF communities. This was largely driven by the abundance of *Fraxinus americana*, which showed the strongest negative correlation (*r* = -0.70), while *Acer rubrum* showed the strongest positive correlation with the PCoA axis (*r* = 0.33; Table S10). Among ECMF genera, *Russula* decreased while *Lactarius*, *Tuber*,* Sarcodon*, and *Tomentella* increased with *F. americana* basal area. Interestingly, *Russula* and *Lactarius* which are both in Russulaceae, showed divergent responses to AM tree composition. While these fungal genera can differ in the production of extramatrical mycelium, both show an affinity for inorganic N, which may be enhanced by AM trees (Avis et al. 2003; Phillips et al. 2013; Suz et al. 2014; Looney et al. 2018). *Fraxinus* litter is even more labile than *Acer* litter (Jacob et al. 2009), which may lead to subtle differences in nutrient cycling within each tree’s respective neighborhood. Although not assayed in our study, soils beneath *Fraxinus* have been shown to exhibit greater phosphorus availability relative to other AM trees (Zheng et al. 2022). Soil phosphorus has been reported to positively influence *Lactarius* and *Tomentella* abundance belowground as well as *Sarcodon* sporocarp production (Taniguchi et al. 2009; Lee et al. 2024; Zavišić et al. 2016), suggesting that nutrient limitation besides N in the region (Vadeboncoeur 2010) may influence ECMF composition. *F. americana* basal area was positively correlated with soil pH (*r* = 0.58; Table S11) which is consistent with their low tolerance for acidic, nutrient poor soils (Burns 1990). Additionally, the ECMF *Tuber* is often most prevalent at higher soil pH (Tedersoo et al. 2014b; Ge et al. 2017) which may partially explain its association with *F. americana* in our study.

### Potential mechanisms driving interactions between ECMF and non-host trees

We hypothesize three potential stabilizing mechanisms (Chesson 2000) where non-host tree diversity may positively influence ECMF diversity. First, we hypothesize that increased AM tree diversity along varying levels of ECM tree dominance likely results in greater spatial heterogeneity of different leaf litter inputs of varying quality (Uriarte et al. 2015). Greater litter diversity can also result in synergistic changes to decomposition (Madritch and Cardinale 2007; Liu et al. 2020), reducing the cycling rates of labile constituents (Grossman et al. 2020), and subsequently influencing soil enzyme activity and ECMF composition (Conn and Dighton 2000). Because communities of ECMF can vary at minute spatial scales (Bruns 1995; Taylor 2002; Tedersoo et al. 2003), increased spatial heterogeneity in litter types and organic substrate availability may partly explain our observations of increased ECMF diversity.

Second, we hypothesize that increases in the phylogenetic diversity of AM trees in mixed AM-ECM forests corresponds to increased root functional diversity (Valverde-Barrantes et al. 2015; Ma et al. 2018). This may drive niche complementarity effects that enhance root foraging efficiency as well as lead to greater depletion of organic and mineral forms of N and phosphorus (Kahmen et al. 2006; Liu et al. 2015, 2018; Valverde-Barrantes et al. 2015; Chen et al. 2016). In our study, AM tree phylogenetic diversity was negatively correlated with soil N, suggesting that greater nutrient foraging efficiency may have depleted pools of soil N and increased the dependency on ECMF for N uptake by ECM trees (Högberg et al. 2003, 2011).

Third, we hypothesize that the rich AM and saprotrophic fungal communities associated with AM trees (Eagar et al. 2022) may alter ECM associations through competition with ECMF for physical access to substrates (Gadgil and Gadgil 1971; Read and Perez-Moreno 2003; Fernandez and Kennedy 2016; Corrales et al. 2018; Bunn et al. 2019). Such interactions may potentially influence the growth and competitive ability of certain ECMF taxa (Baar and Stanton 2000; McHugh and Gehring 2006). ECMF not only compete for growing space and carbon from host plant roots, but also for limiting nutrients in soil (Kennedy and Bruns 2005; Kennedy et al. 2009; Smith and Read 2010; Smith et al. 2023), which may explain reports of antagonisms limiting ECMF co-occurrence (Koide et al. 2005; Lian et al. 2006). Increases in the functional diversity of microbial communities can reduce the performance of the most competitive species, subsequently enhancing taxonomic diversity within individual functional groups (Jousset et al. 2016; Maynard et al. 2017). Therefore, competitive interactions from other fungal trophic guilds may hinder the performance of the most aggressive ECMF and prevent them from competitively excluding other ECMF in mixed AM-ECM forests. These interactions may be further driven by positive relationships between tree diversity and fine root turnover rates (Lei et al. 2012), which may maintain space on ECM fine roots for higher ECMF diversity through an increased probability of new ECM colonization events (Bruns 1995).

### ECM nutrient syndromes predict composition of ECMF and exploration types

Although we did not observe biogeochemical differences that were as strong as expected between AM and ECM-dominated patches (Phillips et al. 2013), soil conditions consistent with an ECM nutrient economy influenced the ECMF taxononomic and functional composition. Most striking were the negative relationships between soil N and the diversity of both ECMF taxa and functional groups. Previous studies have shown that elevated soil N decreases diversity (Högberg et al. 2011; Lilleskov et al. 2019) and alters the composition of ECMF (Arnolds 1991; Lilleskov et al. 2002; Bashian-Victoroff et al. 2025), likely through reduced dependency on extracellular enzymes for N acquisition (Fernandez and See 2025). In our study, soil C: N ratio was positively related to exploration type diversity as well as the relative abundance of mat, medium distance-fringe, and medium distance-smooth exploration type ECMF. Certain genera of ECMF, such as *Craterellus*, *Hydnum*, and *Sarcodon*, responded positively to C: N ratio, with the latter two genera exhibiting a mat-type exploration strategy that can access N from recalcitrant litter like the medium-distance fringe exploration type (Hobbie and Agerer 2010). While the foraging strategy of *Craterellus* is not well defined, sporocarp surveys have reported high abundances in rotting wood (Trappe 2004), suggesting an affinity for high C: N substrates and soils.

However, we also detected positive relationships of soil respiration and pH, which are often higher under AM tree-dominated patches (Phillips et al. 2013; Lang et al. 2020; Zhang et al. 2023), with the diversity of both ECMF and exploration types. Soil respiration is influenced by a variety of processes including fine root abundance, litterfall quantity, and microbial activity (Bowden et al. 1993). In our study, soil respiration was positively correlated with fine root mass (*r* = 0.41) and negatively correlated with forest floor mass (*r* =-0.38; Table S5), suggesting that sites with generally high root mass and high litter turnover rates were more conducive to a high taxonomic and functional ECMF diversity. However, the degree to which ECMF contributed to or were influenced by soil respiration rates in our study remains unknown.

### Implications of non-host trees for temperate AM-ECM forests

The potential for non-host effects of AM trees on ECM fungi is consistent with the idea that spillover effects of dominant trees (Eagar et al. 2025) are an important regulator of the fungal communities experienced by nearby plants, including ECMF communities, in mixed temperate forests. The heterogeneous patchwork of AM and ECM trees in forests may create a mosaic of ECMF niche space through complex interactions between leaf litter inputs, belowground processes, and edaphic factors (Bruns 1995). In ECM-dominated forest stands, associated litter inputs (Phillips et al. 2013) may foster a narrow niche breadth where specialized ECMF capable of accessing limiting nutrients from recalcitrant substrates dominate communities (Hobbie and Agerer 2010; Pellitier and Zak 2018). The presence of a diverse assemblage of AM trees may enhance the breadth of available niche space for ECMF, potentially through interactions with their labile leaf litter, roots, and associated fungal communities. However, global change is increasing AM tree dominance in mixed AM-ECM forests (Averill et al. 2018; Jo et al. 2019), of which modest increases may actually enhance ECMF diversity in some cases, although a transition to total AM dominance would likely lead to declines of ECMF across the landscape (Eagar et al. 2023; Cortese et al. 2023; Cortese and Horton 2024).

## Conclusions

We found evidence that AM trees influence ECMF communities, suggesting that non-host plant composition may be important in maintaining niche space to support mutualist diversity. Ultimately, we suspect that our three hypothesized mechanisms above do not exist in isolation, and feedbacks between litter chemistry, nutrient availability, root production, and interguild competition that influence ECMF composition likely co-occur in parallel in mixed AM-ECM forests. Accounting for non-host vegetation is therefore necessary to better understand the linkages between above- and below-ground biota. Thus, as tree communities increasingly shift to AM tree dominance due to global change, there may be some positive effects on ECMF diversity due to non-host interactions. However, these relationships are likely conditional on the maintenance of ECM trees, and the complete replacement by AM trees will likely lead to diminished ECM fungal diversity in mixed AM-ECM forests.

## Electronic Supplementary Material

Below is the link to the electronic supplementary material.

Supplementary Material 1

## Data Availability

All sequence data can be found in the Sequence Read Archive (PRJNA837524) while all tree community and soil variable data used in this study can be found in the Figshare repository (https://doi.org/10.6084/m9.figshare.19795558.v1).
