# Supplementary material for: Ectomycorrhizal fungal diversity may be influenced by arbuscular mycorrhizal trees in mixed temperate forests
Source: Mycorrhiza. 2026 May 11;36(3):24. doi: 10.1007/s00572-026-01267-2 (PMC13158259; doi:10.1007/s00572-026-01267-2)
Supplement: Supplementary file 1 — Supplementary Material 1 [file 572_2026_1267_MOESM1_ESM.docx]

SUPPORTING INFORMATION

SUPPORTING METHODS 1

*Calculation of tree diversity variables*

For each plot, we calculated Hill *^0^D* (species richness) and Hill *^2^D* (inverse Simpson index) diversity indices for AM trees alone, ECM trees alone, and combined ECM and AM trees (Hill 1973; Chao et al. 2014). We used both basal area and stem counts for calculation of Hill *^2^D* because they were not highly correlated (*r* <0.6) and represent measures that give more weight to overstory and understory dominance of each tree species, respectively (Mueller-Dombois and Ellenberg 1974). For indices of tree phylogenetic diversity, we generated a phylogenetic tree in R version 4.2.2 (R Core Team 2022) for all tree species sampled in our plots from the megatree created by Smith and Brown (2018) using the package *rtrees* (Li 2023; Fig. S1)*.* Summed branch lengths from the phylogenetic tree (Faith 1992) were used to calculate plot-level phylogenetic diversity (phylo-Hill *^0^D* and phylo-Hill *^2^D,* derived from basal area) for AM, ECM, and combined AM-ECM tree communities with the package *adiv* (Pavoine 2020). To generate phylogenetically-weighted variables representing tree community composition, we then used the package *betapart* (Baselga and Orme 2012) to calculate plot-level pairwise Sørensen phylogenetic dissimilarity for AM, ECM, and combined AM and ECM tree communities, which we then converted into phylogenetic eigenvectors using principal coordinate analysis (PCoA) with the package *vegan* (Oksanen 2019). Summaries of tree taxonomic and phylogenetic diversity variables (except for phylogenetic eigenvectors) can be found in the Supporting Information (Table S2).

SUPPORTING METHODS 2

*Modeling ECMF diversity responses*

*First*, we used linear mixed-effects regressions, with site (Shingle Shanty, Huntington Forest, Deer Leap) as a random effect, with the package *lme4* (Bates et al. 2014). We constructed separate global models containing each suite of AM tree diversity (Table S2), ECM tree diversity (Table S2), combined AM-ECM tree diversity (Table S2), tree community abundance by mycorrhizal type (AM, ECM, and combined AM-ECM; Table S3), and soil variables (Table S4) to identify the most informative variables from each of the five variable suites. Among tree diversity variables, we included Hill *^0^D* (species richness), Hill *^2^D* (inverse Simpson’s diversity index) using basal area and stem counts as separate abundance measures, phylogenetic Hill *^0^D* (Faith’s distance; Faith 1992), as well as phylogenetic Hill *^2^D* for AM, ECM, and combined AM-ECM tree communities. *Second*, we tested for multicollinearity within each variable suite by calculating the variance inflation factor (VIF) of each global model using the package *car* (Fox and Weisberg 2018). If models contained variables with VIF ≥ 10, we sequentially removed individual candidate variables with the highest VIF, refit the model, and then repeated until VIF < 10 for all remaining variables (Borcard et al. 2018). *Third*, we conducted *unsupervised* variable selection on each global model containing separate suites of variables (e.g., AM tree diversity, ECM tree diversity, combined AM-ECM tree diversity, tree community abundance by mycorrhizal type, and soil variables), and ranked the models with corrected Akaike’s Information Criterion (AICc) using the dredge() function with the package *MuMIn* (Bartoń 2023)*.* We then selected all variables included in each top AICc-ranked model, representing the most informative predictors from each variable suite, to be included in the next model selection step. *Fourth*, we conducted *supervised* variable selection by fitting separate linear mixed-effects models containing variables individually, or in biologically relevant combinations. For this step we specified models with each unique combination of soil variables but only allowed one tree abundance and one tree diversity variable in the same model. We specified interaction terms between soil variables if multiple were included in the same model, as well as interactions between tree abundance and diversity variables. However, to avoid uninterpretable higher-order interactions, we did not specify interactions between soil variables and tree diversity or abundance variables. We also specified a null model only containing the intercept random effect terms. We then ranked all candidate models with AICc using the package *AICcmodavg* (Mazerolle 2023) to identify the most parsimonious models explaining each ECMF taxonomic diversity response. We checked the residuals of each top model to ensure the assumptions of normality and homogeneity of variance were fulfilled. To assess overall model fit, we calculated conditional R^2^ values for each top-ranked model with the package *MuMIn.* *Fifth*, we removed all models containing variables that were collinear (VIF≥ 10) to those contained in the top-ranked model. We also removed any candidate models <2 ΔAICc containing uninformative parameters that were extensions of the top model (*sensu* Arnold 2010). The remaining set of models was then used to model-average ECMF diversity responses to each variable using the package *AICcmodavg*. We assessed the significance of each variable by examining the 95% confidence intervals; if confidence intervals did *not* intersect zero, then the variable was considered significant.

SOURCES

**Bartoń K** (**2023**) MuMIn: Multi-Model Inference. R package ver. 1.47.5.

**Baselga A, Orme CDL** (**2012**) betapart: an R package for the study of beta diversity. *Methods in Ecology and Evolution* **3**: 808–812.

**Bates D, Mächler M, Bolker B, Walker S** (**2014**) Fitting Linear Mixed-Effects Models using lme4.

**Fox J, Weisberg S** (**2018**) *An R Companion to Applied Regression*. SAGE Publications.

**Faith DP** (**1992**) Conservation evaluation and phylogenetic diversity. *Biological Conservation* **61**: 1–10.

**Hill MO** (**1973**) Diversity and Evenness: A Unifying Notation and Its Consequences. *Ecology* **54**: 427–432.

**Mazerolle MJ** (**2023**) AICcmodavg: Model Selection and Multimodel Inference Based on (Q)AIC(c) ver. 2.3-3.

**Mueller-Dombois D, Ellenberg H** (**1974**) Aims and methods of vegetation ecology.

**Oksanen J** (**2019**) Package ‘Vegan’.

**Pavoine S** (**2020**) adiv: An r package to analyse biodiversity in ecology. *Methods in Ecology and Evolution* **11**: 1106–1112.

**Smith SA, Brown JW** (**2018**) Constructing a broadly inclusive seed plant phylogeny. *American Journal of Botany* **105**: 302–314.

**Table S1** Percent basal area (± SE) of arbuscular mycorrhizal (AM) and ectomycorrhizal (ECM) trees sampled from 71 plots across three sites in the Adirondack Mountains, NY (2017)

|  |  |  |  |
| --- | --- | --- | --- |
| Tree species | Deer Leap | Huntington Forest | Shingle Shanty |
| *Acer saccharum* (AM) | 35.51 ± 4.31 | 33.39 ± 4.38 | 16.96 ± 4.05 |
| *Acer rubrum* (AM) | 1.26 ± 0.58 | 0.76 ± 0.52 | 21.75 ± 5.05 |
| *Acer pensylvanicum* (AM) | 0.12 ± 0.05 | 0.46 ± 0.27 | 0.60 ± 0.22 |
| *Fagus grandifolia* (ECM) | 19.57 ± 2.79 | 31.79 ± 4.30 | 39.24 ± 3.71 |
| *Ostrya virginiana* (ECM) | 0.86 ± 0.35 | 0.07 ± 0.07 | 0.00 ± 0.00 |
| *Tsuga canadensis* (ECM) | 1.34 ± 0.50 | 11.46 ± 2.84 | 5.15 ± 2.83 |
| *Pinus strobus* (ECM) | 10.19 ± 4.03 | 0.00 ± 0.00 | 0.00 ± 0.00 |
| *Pinus resinosa* (ECM) | 0.22 ± 0.22 | 0.00 ± 0.00 | 0.00 ± 0.00 |
| *Picea rubens* (ECM) | 0.00 ± 0.00 | 3.26 ± 1.16 | 6.24 ± 1.23 |
| *Betula alleghaniensis* (ECM) | 9.67 ± 2.45 | 17.48 ± 4.18 | 10.02 ± 2.61 |
| *Betula papyrifera* (ECM) | 1.59 ± 0.73 | 0.05 ± 0.05 | 0.00 ± 0.00 |
| *Hamamelis virginiana* (AM) | 0.02 ± 0.01 | 0.00 ± 0.00 | 0.00 ± 0.00 |
| *Quercus rubra* (ECM) | 10.07 ± 3.32 | 0.00 ± 0.00 | 0.00 ± 0.00 |
| *Quercus alba* (ECM) | 0.80 ± 0.38 | 0.00 ± 0.00 | 0.00 ± 0.00 |
| *Fraxinus americana* (AM) | 6.55 ± 1.61 | 1.29 ± 0.47 | 0.04 ± 0.04 |
| *Ulmus americana* (AM) | 0.01 ± 0.00 | 0.00 ± 0.00 | 0.00 ± 0.00 |
| *Populus grandidentata* (ECM) | 0.44 ± 0.36 | 0.00 ± 0.00 | 0.00 ± 0.00 |
| *Tilia americana* (ECM) | 1.77 ± 0.76 | 0.00 ± 0.00 | 0.00 ± 0.00 |
|  |  |  |  |


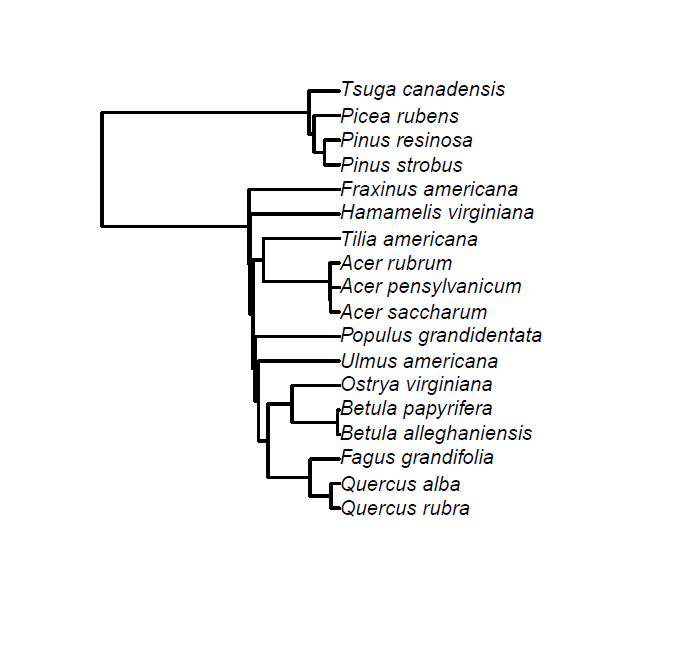


**Fig. S1** Phylogenetic tree (Li, 2023) of AM and ECM tree species present in study used for calculating plot-level AM, ECM, and combined AM-ECM phylogenetic diversity from the Adirondack Mountains, USA (2017)

**Table S2** Plot ectomycorrhizal (ECM), arbuscular mycorrhizal (AM), and combined AM-ECM tree diversity variable summaries used in models of ECMF taxonomic and functional diversity from the Adirondack Mountains, USA (2017)

| Variable | Mean ± SE |
| --- | --- |
| AM tree phylogenetic Hill *^0^D* | 456.14 ± 10.03 |
| AM tree phylogenetic Hill *^2^D* | 404.28 ± 2.66 |
| ECM tree phylogenetic Hill *^0^D* | 775.73 ± 16.08 |
| ECM tree phylogenetic Hill *^2^D* | 520.89 ± 11.37 |
| Combined ECM and AM tree phylogenetic Hill *^0^D* | 957.84 ± 19.24 |
| Combined ECM and AM tree phylogenetic Hill *^2^D* | 545.46 ± 10.19 |
| AM tree Hill *^0^D* | 2.21 ± 0.11 |
| AM stem Hill *^2^D* | 1.57 ± 0.06 |
| AM basal area Hill *^2^D* | 1.16 ± 0.04 |
| ECM tree Hill *^0^D* | 3.49 ± 0.16 |
| ECM stem Hill *^2^D* | 1.66 ± 0.06 |
| ECM basal area Hill *^2^D* | 2.17 ± 0.11 |
| Combined ECM and AM tree Hill *^0^D* | 5.69 ± 0.22 |
| Combined ECM and AM basal area Hill *^2^D* | 2.82 ± 0.11 |
| Combined ECM and AM stem Hill *^2^D* | 2.23 ± 0.10 |
|  |  |

**Table S3** Plot tree community abundance variable summaries used in models of ECMF taxonomic and functional diversity from the Adirondack Mountains, USA (2017)

| Variable | Mean ± SE |
| --- | --- |
| Combined ECM and AM stems | 1595.29 ± 55.09 |
| Combined ECM and AM basal area (m^2^) | 2.55 ± 0.07 |
| % Conifer basal area | 13.05 ± 2.00 |
| % ECM stems | 84.08 ± 1.49 |
| % ECM basal area | 60.46 ± 2.48 |
| ECM importance value (IV) | 144.54 ± 3.31 |
| Total ECM basal area (m^2^) | 1.54 ± 0.08 |
| Total ECM stems | 1365.20 ± 61.18 |
| % AM stems | 15.92 ± 1.49 |
| % AM basal area | 39.54 ± 2.48 |
| AM importance value (IV) | 55.46 ± 3.31 |
| Total AM basal area (m^2^) | 1.01 ± 0.07 |
| Total AM stems | 230.10 ± 20.01 |

**Table S4** Plot soil variable summaries from the Adirondack Mountains, USA (2017)

| Variable | Mean ± SE |
| --- | --- |
| pH | 4.27 ± 0.09 |
| % Carbon | 17.6 ± 1.50 |
| % Nitrogen | 0.83 ± 0.06 |
| C:N ratio | 20.40 ± 0.59 |
| NH_4_^+^ (μg g^-1^) | 24.45 ± 2.20 |
| N mineralization (μg g^-1^ day^-1^) | 7.56 ± 0.50 |
| NO_3_^-^ (μg g^-1^) | 10.86 ± 1.20 |
| Nitrification (μg g^-1^ day^-1^) | 4.09 ± 0.38 |
| Total inorganic nitrogen (μg g^-1^) | 35.32 ± 2.83 |
| Forest floor (Oi + Oe) mass (g m^-3^) | 945.72 ± 36.03 |
| Fine root biomass (kg m^-3^) | 2.31 ± 0.08 |
| Soil respiration (μmol CO_2_ m^-2^s^-1^) | 12.21 ± 0.43 |
|  |  |

**Table S5** Soil variable Pearson correlations with tree diversity and abundance variables from the Adirondack Mountains, USA (2017). Significant (*p*<0.05) correlations are indicated in bold text

|  | NH_4_^+^ | N min. | NO_3_^-^ | Nitrification | Inorg. N | Forest floor mass | Fine root mass | Soil resp. | pH | % C | % N | C:N ratio |
| --- | --- | --- | --- | --- | --- | --- | --- | --- | --- | --- | --- | --- |
| AM tree phylo. *^0^D* | -0.04 | **-0.25** | -0.07 | 0.02 | -0.06 | **0.31** | -0.17 | **-0.27** | **0.57** | **-0.37** | **-0.39** | -0.08 |
| AM tree phylo. *^2^D* | -0.10 | -0.19 | -0.03 | 0.02 | -0.09 | 0.22 | **-0.32** | **-0.30** | **0.47** | **-0.35** | **-0.37** | -0.03 |
| AM tree *^0^D* | 0.20 | -0.11 | -0.02 | -0.02 | 0.15 | 0.07 | 0.07 | -0.12 | **0.40** | -0.16 | -0.19 | 0.04 |
| AM stem *^2^D* | **0.30** | 0.10 | -0.07 | -0.06 | 0.20 | **-0.26** | 0.18 | 0.10 | -0.02 | 0.20 | 0.17 | 0.12 |
| AM basal area *^2^D* | 0.12 | -0.16 | 0.07 | -0.18 | 0.12 | -0.01 | -0.05 | -0.05 | 0.06 | -0.09 | -0.12 | 0.14 |
| ECM tree phylo. *^0^D* | 0.17 | 0.02 | -0.01 | -0.06 | 0.12 | 0.24 | -0.04 | -0.08 | 0.03 | 0.13 | 0.08 | **0.31** |
| ECM tree phylo *^2^D* | 0.18 | 0.10 | -0.02 | -0.12 | 0.13 | 0.20 | -0.20 | 0.05 | 0.05 | **0.25** | 0.19 | **0.28** |
| ECM tree *^0^D* | -0.04 | **-0.33** | -0.15 | -0.18 | -0.10 | **0.53** | **-0.26** | **-0.42** | **0.45** | -0.21 | -0.23 | 0.05 |
| ECM stem *^2^D* | **0.37** | -0.08 | 0.11 | -0.17 | **0.33** | 0.05 | -0.02 | -0.13 | 0.02 | 0.15 | 0.09 | **0.27** |
| ECM basal area *^2^D* | -0.05 | -0.06 | 0.11 | 0.03 | 0.01 | **0.32** | -0.22 | **-0.27** | **0.48** | -0.17 | -0.19 | -0.04 |
| AM + ECM tree phylo *^0^D* | 0.00 | -0.03 | -0.05 | -0.12 | -0.02 | **0.30** | **-0.24** | -0.07 | 0.05 | 0.14 | 0.09 | 0.21 |
| AM + ECM tree phylo *^2^D* | 0.13 | -0.10 | -0.05 | -0.04 | 0.08 | **0.36** | -0.12 | -0.20 | **0.29** | -0.07 | -0.12 | 0.23 |
| AM + ECM tree *^0^D* | 0.07 | **-0.29** | -0.12 | -0.14 | 0.00 | **0.42** | -0.15 | **-0.37** | **0.52** | -0.23 | **-0.26** | 0.06 |
| AM + ECM basal area *^2^D* | -0.08 | -0.14 | 0.06 | -0.01 | -0.04 | **0.30** | -0.16 | **-0.28** | **0.41** | -0.11 | -0.12 | -0.05 |
| AM + ECM stem *^2^D* | **0.32** | -0.09 | 0.10 | -0.18 | **0.29** | 0.16 | 0.03 | -0.18 | 0.20 | 0.09 | 0.06 | 0.16 |
| Stem density | **0.35** | **0.50** | **0.37** | 0.23 | **0.43** | **-0.32** | 0.18 | **0.35** | **-0.55** | 0.16 | 0.06 | **0.37** |
| Basal area | 0.00 | **-0.32** | -0.09 | -0.17 | -0.04 | **0.38** | **-0.24** | -0.20 | **0.29** | -0.10 | -0.11 | 0.03 |
| % AM stems | 0.12 | -0.05 | 0.04 | -0.02 | 0.11 | **0.24** | 0.03 | **-0.25** | **0.47** | -0.16 | -0.15 | -0.13 |
| % AM basal area | -0.09 | 0.01 | 0.05 | 0.05 | -0.05 | -0.15 | 0.14 | 0.01 | 0.14 | -0.08 | -0.03 | **-0.34** |
| AM importance value | -0.01 | -0.01 | 0.05 | 0.03 | 0.02 | 0.00 | 0.12 | -0.10 | **0.32** | -0.13 | -0.08 | **-0.31** |
| AM basal area | -0.13 | -0.08 | 0.03 | 0.01 | -0.08 | -0.08 | 0.07 | -0.01 | 0.19 | -0.07 | -0.02 | **-0.34** |
| % ECM stems | -0.12 | 0.05 | -0.04 | 0.02 | -0.11 | **-0.24** | -0.03 | **0.25** | **-0.47** | 0.16 | 0.15 | 0.13 |
| % ECM basal area | 0.09 | -0.01 | -0.05 | -0.05 | 0.05 | 0.15 | -0.14 | -0.01 | -0.14 | 0.08 | 0.03 | **0.34** |
| ECM importance value | 0.01 | 0.01 | -0.05 | -0.03 | -0.02 | 0.00 | -0.12 | 0.10 | **-0.32** | 0.13 | 0.08 | **0.31** |
| ECM basal area | 0.11 | -0.19 | -0.10 | -0.15 | 0.04 | **0.39** | **-0.25** | -0.16 | 0.08 | -0.02 | -0.08 | **0.31** |
| ECM dominance | 0.00 | -0.04 | 0.02 | -0.07 | 0.01 | 0.07 | -0.07 | 0.07 | **-0.25** | 0.21 | 0.15 | **0.36** |
| NH_4_^+^ |  | **0.49** | **0.34** | -0.01 | **0.92** | -0.16 | 0.13 | 0.13 | -0.13 | **0.26** | 0.18 | **0.49** |
| N_min | **0.49** |  | **0.59** | **0.66** | **0.63** | -0.22 | **0.29** | **0.27** | -0.18 | 0.16 | 0.08 | 0.22 |
| NO_3_^-^ | **0.34** | **0.59** |  | **0.50** | **0.69** | -0.21 | 0.14 | 0.21 | **-0.25** | 0.05 | -0.05 | **0.38** |
| Nitrification | -0.01 | **0.66** | **0.50** |  | 0.21 | -0.04 | 0.09 | 0.09 | 0.08 | -0.16 | -0.18 | -0.07 |
| Total inorganic N | **0.92** | **0.63** | **0.69** | 0.21 |  | -0.21 | 0.16 | 0.19 | -0.21 | 0.22 | 0.12 | **0.54** |
| Forest floor mass | -0.16 | -0.22 | -0.21 | -0.04 | -0.21 |  | -0.18 | **-0.38** | **0.48** | **-0.27** | -0.22 | -0.22 |
| Fine root mass | 0.13 | **0.29** | 0.14 | 0.09 | 0.16 | -0.18 |  | **0.41** | -0.18 | 0.19 | 0.19 | 0.06 |
| Soil respiration | 0.13 | **0.27** | 0.21 | 0.09 | 0.19 | **-0.38** | **0.41** |  | **-0.49** | **0.46** | **0.43** | **0.36** |
| pH | -0.13 | -0.18 | **-0.25** | 0.08 | -0.21 | **0.48** | -0.18 | **-0.49** |  | **-0.52** | **-0.45** | **-0.48** |
| % C | **0.26** | 0.16 | 0.05 | -0.16 | 0.22 | **-0.27** | 0.19 | **0.46** | **-0.52** |  | **0.97** | **0.49** |
| % N | 0.18 | 0.08 | -0.05 | -0.18 | 0.12 | -0.22 | 0.19 | **0.43** | **-0.45** | 0.97 |  | **0.30** |
| C:N ratio | **0.49** | 0.22 | **0.38** | -0.07 | **0.54** | -0.22 | 0.06 | **0.36** | **-0.48** | 0.49 | **0.30** |  |
|  |  |  |  |  |  |  |  |  |  |  |  |  |

**Table S6** List of ectomycorrhizal fungi (ECMF) sequenced from roots and soils from 72 plots across three sites in the Adirondack Mountains, NY (2017)

|  |  |  |  |  |  |  |
| --- | --- | --- | --- | --- | --- | --- |
|  | Deer Leap (% ± SE) | | Huntington Forest (% ± SE) | | Shingle Shanty (% ± SE) | |
| ECMF Genus | Root | Soil | Root | Soil | Root | Soil |
| *Amanita* | 0.15 ± 0.06 | 0.92 ± 0.58 | 0.98 ± 0.23 | 3.17 ± 0.72 | 1.85 ± 0.52 | 3.17 ± 0.72 |
| *Amphinema* | 0.11 ± 0.09 | 0.24 ± 0.13 | 0.00 ± 0.00 | 0.01 ± 0.01 | 0.00 ± 0.00 | 0.01 ± 0.01 |
| *Boletus* | 0.01 ± 0.01 | 0.03 ± 0.02 | 0.12 ± 0.12 | 0.00 ± 0.00 | 0.00 ± 0.00 | 0.00 ± 0.00 |
| *Byssocorticium* | 0.00 ± 0.00 | 0.07 ± 0.04 | 0.01 ± 0.01 | 0.03 ± 0.02 | 0.00 ± 0.00 | 0.03 ± 0.02 |
| *Cantharellus* | 0.00 ± 0.00 | 0.00 ± 0.00 | 0.00 ± 0.00 | 0.00 ± 0.00 | 0.00 ± 0.00 | 0.00 ± 0.00 |
| *Cenococcum* | 1.81 ± 0.66 | 1.11 ± 0.23 | 1.35 ± 0.41 | 1.21 ± 0.31 | 2.33 ± 0.48 | 1.21 ± 0.31 |
| *Clavulina* | 0.23 ± 0.23 | 0.63 ± 0.61 | 0.71 ± 0.65 | 2.59 ± 1.45 | 0.05 ± 0.03 | 2.59 ± 1.45 |
| *Coltricia* | 0.03 ± 0.02 | 0.04 ± 0.03 | 0.00 ± 0.00 | 0.00 ± 0.00 | 0.00 ± 0.00 | 0.00 ± 0.00 |
| *Cortinarius* | 5.83 ± 2.60 | 6.34 ± 2.90 | 1.11 ± 0.36 | 2.73 ± 0.82 | 2.63 ± 1.28 | 2.73 ± 0.82 |
| *Craterellus* | 0.72 ± 0.58 | 0.81 ± 0.69 | 0.33 ± 0.25 | 0.54 ± 0.48 | 0.00 ± 0.00 | 0.54 ± 0.48 |
| *Densospora* | 0.00 ± 0.00 | 0.00 ± 0.00 | 0.00 ± 0.00 | 0.00 ± 0.00 | 0.00 ± 0.00 | 0.00 ± 0.00 |
| *Elaphomyces* | 0.01 ± 0.01 | 0.15 ± 0.15 | 0.01 ± 0.01 | 0.52 ± 0.23 | 0.01 ± 0.01 | 0.52 ± 0.23 |
| *Genea* | 0.00 ± 0.00 | 0.02 ± 0.02 | 0.02 ± 0.02 | 0.01 ± 0.01 | 0.00 ± 0.00 | 0.01 ± 0.01 |
| *Gyroporus* | 0.00 ± 0.00 | 0.00 ± 0.00 | 0.00 ± 0.00 | 0.01 ± 0.01 | 0.00 ± 0.00 | 0.01 ± 0.01 |
| *Hebeloma* | 0.18 ± 0.15 | 0.20 ± 0.12 | 0.16 ± 0.09 | 0.29 ± 0.20 | 0.03 ± 0.03 | 0.29 ± 0.20 |
| *Helvellosebacina* | 0.08 ± 0.05 | 0.40 ± 0.20 | 0.00 ± 0.00 | 0.00 ± 0.00 | 0.00 ± 0.00 | 0.00 ± 0.00 |
| *Humaria* | 0.08 ± 0.04 | 0.05 ± 0.04 | 0.00 ± 0.00 | 0.00 ± 0.00 | 0.00 ± 0.00 | 0.00 ± 0.00 |
| *Hydnobolites* | 0.01 ± 0.01 | 0.11 ± 0.07 | 0.00 ± 0.00 | 0.00 ± 0.00 | 0.00 ± 0.00 | 0.00 ± 0.00 |
| *Hydnotrya* | 0.00 ± 0.00 | 0.00 ± 0.00 | 0.00 ± 0.00 | 0.03 ± 0.02 | 0.00 ± 0.00 | 0.03 ± 0.02 |
| *Hydnum* | 0.08 ± 0.06 | 0.09 ± 0.06 | 0.10 ± 0.10 | 0.26 ± 0.26 | 0.00 ± 0.00 | 0.26 ± 0.26 |
| *Hygrophorus* | 0.32 ± 0.18 | 1.89 ± 1.56 | 0.01 ± 0.01 | 0.01 ± 0.01 | 0.00 ± 0.00 | 0.01 ± 0.01 |
| *Hymenogaster* | 0.03 ± 0.03 | 0.17 ± 0.10 | 0.05 ± 0.05 | 0.00 ± 0.00 | 0.00 ± 0.00 | 0.00 ± 0.00 |
| *Hysterangium* | 0.00 ± 0.00 | 0.00 ± 0.00 | 0.00 ± 0.00 | 0.00 ± 0.00 | 0.00 ± 0.00 | 0.00 ± 0.00 |
| *Imleria* | 0.00 ± 0.00 | 0.02 ± 0.02 | 0.05 ± 0.03 | 0.38 ± 0.15 | 0.16 ± 0.06 | 0.38 ± 0.15 |
| *Inocybe* | 0.58 ± 0.26 | 2.85 ± 1.25 | 1.87 ± 0.63 | 6.01 ± 1.63 | 0.50 ± 0.21 | 6.01 ± 1.63 |
| *Laccaria* | 0.07 ± 0.03 | 0.42 ± 0.25 | 0.06 ± 0.04 | 0.33 ± 0.12 | 0.02 ± 0.02 | 0.33 ± 0.12 |
| *Lactarius* | 1.43 ± 0.39 | 0.70 ± 0.21 | 1.22 ± 0.35 | 1.12 ± 0.25 | 0.27 ± 0.11 | 1.12 ± 0.25 |
| *Lactifluus* | 0.03 ± 0.03 | 0.08 ± 0.08 | 0.00 ± 0.00 | 0.01 ± 0.01 | 0.11 ± 0.10 | 0.01 ± 0.01 |
| *Leccinum* | 0.00 ± 0.00 | 0.03 ± 0.02 | 0.14 ± 0.13 | 0.10 ± 0.07 | 0.01 ± 0.01 | 0.10 ± 0.07 |
| *Leotia* | 0.00 ± 0.00 | 0.00 ± 0.00 | 0.00 ± 0.00 | 0.00 ± 0.00 | 0.00 ± 0.00 | 0.00 ± 0.00 |
| *Membranomyces* | 0.19 ± 0.13 | 0.21 ± 0.10 | 0.04 ± 0.02 | 0.19 ± 0.09 | 0.05 ± 0.04 | 0.19 ± 0.09 |
| *Naucoria* | 0.02 ± 0.02 | 0.06 ± 0.06 | 0.00 ± 0.00 | 0.01 ± 0.01 | 0.00 ± 0.00 | 0.01 ± 0.01 |
| *Octaviania* | 0.00 ± 0.00 | 0.00 ± 0.00 | 0.00 ± 0.00 | 0.00 ± 0.00 | 0.00 ± 0.00 | 0.00 ± 0.00 |
| *Otidea* | 0.04 ± 0.04 | 0.16 ± 0.11 | 0.00 ± 0.00 | 0.00 ± 0.00 | 0.00 ± 0.00 | 0.00 ± 0.00 |
| *Pachyphlodes* | 0.21 ± 0.11 | 0.59 ± 0.18 | 0.10 ± 0.07 | 0.62 ± 0.35 | 0.00 ± 0.00 | 0.62 ± 0.35 |
| *Paxillus* | 0.00 ± 0.00 | 0.04 ± 0.03 | 0.00 ± 0.00 | 0.03 ± 0.01 | 0.00 ± 0.00 | 0.03 ± 0.01 |
| *Phaeocollybia* | 0.00 ± 0.00 | 0.00 ± 0.00 | 0.00 ± 0.00 | 0.00 ± 0.00 | 0.07 ± 0.06 | 0.00 ± 0.00 |
| *Phaeohelotium* | 0.58 ± 0.22 | 0.03 ± 0.01 | 2.87 ± 0.86 | 0.14 ± 0.07 | 0.82 ± 0.42 | 0.14 ± 0.07 |
| *Piloderma* | 5.64 ± 1.94 | 6.63 ± 3.08 | 0.61 ± 0.38 | 0.61 ± 0.34 | 1.78 ± 1.16 | 0.61 ± 0.34 |
| *Protoglossum* | 0.00 ± 0.00 | 0.25 ± 0.15 | 0.00 ± 0.00 | 0.00 ± 0.00 | 0.00 ± 0.00 | 0.00 ± 0.00 |
| *Pseudotomentella* | 0.17 ± 0.13 | 0.08 ± 0.04 | 0.06 ± 0.05 | 0.02 ± 0.02 | 0.02 ± 0.02 | 0.02 ± 0.02 |
| *Ramaria* | 0.00 ± 0.00 | 0.03 ± 0.03 | 0.00 ± 0.00 | 0.00 ± 0.00 | 0.00 ± 0.00 | 0.00 ± 0.00 |
| *Rhizopogon* | 0.00 ± 0.00 | 0.00 ± 0.00 | 0.00 ± 0.00 | 0.00 ± 0.00 | 0.00 ± 0.00 | 0.00 ± 0.00 |
| *Rhodoscypha* | 0.00 ± 0.00 | 0.00 ± 0.00 | 0.02 ± 0.02 | 0.01 ± 0.01 | 0.00 ± 0.00 | 0.01 ± 0.01 |
| *Russula* | 4.73 ± 1.23 | 9.17 ± 2.18 | 7.95 ± 1.72 | 11.91 ± 2.23 | 8.96 ± 1.45 | 11.91 ± 2.23 |
| *Sarcodon* | 1.51 ± 1.51 | 0.49 ± 0.49 | 0.00 ± 0.00 | 0.00 ± 0.00 | 0.00 ± 0.00 | 0.00 ± 0.00 |
| *Scabropezia* | 0.00 ± 0.00 | 0.11 ± 0.11 | 0.00 ± 0.00 | 0.00 ± 0.00 | 0.00 ± 0.00 | 0.00 ± 0.00 |
| *Scleroderma* | 0.00 ± 0.00 | 0.00 ± 0.00 | 0.00 ± 0.00 | 0.01 ± 0.01 | 0.00 ± 0.00 | 0.01 ± 0.01 |
| *Sebacina* | 0.83 ± 0.24 | 1.70 ± 0.39 | 1.06 ± 0.27 | 2.67 ± 0.75 | 0.98 ± 0.34 | 2.67 ± 0.75 |
| *Suillus* | 0.12 ± 0.09 | 0.25 ± 0.11 | 0.00 ± 0.00 | 0.00 ± 0.00 | 0.00 ± 0.00 | 0.00 ± 0.00 |
| *Tarzetta* | 0.00 ± 0.00 | 0.00 ± 0.00 | 0.00 ± 0.00 | 0.00 ± 0.00 | 0.00 ± 0.00 | 0.00 ± 0.00 |
| *Thelephora* | 0.01 ± 0.01 | 0.03 ± 0.03 | 0.11 ± 0.06 | 0.16 ± 0.07 | 0.05 ± 0.05 | 0.16 ± 0.07 |
| *Tomentella* | 1.28 ± 0.32 | 1.16 ± 0.24 | 0.74 ± 0.24 | 0.72 ± 0.13 | 0.52 ± 0.28 | 0.72 ± 0.13 |
| *Tomentellopsis* | 0.00 ± 0.00 | 0.01 ± 0.01 | 0.00 ± 0.00 | 0.01 ± 0.01 | 0.00 ± 0.00 | 0.01 ± 0.01 |
| *Tremellodendropsis* | 0.00 ± 0.00 | 0.07 ± 0.05 | 0.00 ± 0.00 | 0.00 ± 0.00 | 0.00 ± 0.00 | 0.00 ± 0.00 |
| *Tricholoma* | 2.01 ± 1.64 | 2.41 ± 1.37 | 0.02 ± 0.02 | 0.15 ± 0.10 | 0.00 ± 0.00 | 0.15 ± 0.10 |
| *Trichophaea* | 0.00 ± 0.00 | 0.01 ± 0.00 | 0.00 ± 0.00 | 0.00 ± 0.00 | 0.00 ± 0.00 | 0.00 ± 0.00 |
| *Tuber* | 0.11 ± 0.05 | 0.05 ± 0.03 | 0.17 ± 0.09 | 0.10 ± 0.06 | 0.01 ± 0.01 | 0.10 ± 0.06 |
| *Tylopilus* | 0.00 ± 0.00 | 0.01 ± 0.01 | 0.24 ± 0.16 | 0.09 ± 0.05 | 0.01 ± 0.01 | 0.09 ± 0.05 |
| *Wilcoxina* | 0.05 ± 0.03 | 0.02 ± 0.01 | 0.00 ± 0.00 | 0.00 ± 0.00 | 0.00 ± 0.00 | 0.00 ± 0.00 |
| *Xanthoconium* | 0.01 ± 0.01 | 0.00 ± 0.00 | 0.04 ± 0.03 | 0.13 ± 0.05 | 0.04 ± 0.02 | 0.13 ± 0.05 |
| *Xerocomellus* | 0.00 ± 0.00 | 0.00 ± 0.00 | 0.00 ± 0.00 | 0.08 ± 0.03 | 0.00 ± 0.00 | 0.08 ± 0.03 |
| *Xerocomus* | 0.00 ± 0.00 | 0.07 ± 0.04 | 0.10 ± 0.10 | 0.03 ± 0.01 | 0.00 ± 0.00 | 0.03 ± 0.01 |
|  |  |  |  |  |  |  |

**Table S7** AICc table showing null and top-ranked models used for model-averaged predicted responses of soil and root ECMF taxonomic diversity to tree abundance and diversity, as well as soil variables from the Adirondack Mountains, USA (2017)

|  | **Model^ab^** | ***K*^c^** | **logLik^d^** | **AICc** | **ΔAICc** | ***W_i_*^e^** | **Cond. R^2^** |
| --- | --- | --- | --- | --- | --- | --- | --- |
| Soil ECMF genus *^0^D* | % ECM BA + AM tree phylo. *^2^D* + NO_3_^-^ | 6 | -161.03 | 335.45 | 0.00 | 0.52 | 0.48 |
|  | % ECM BA × AM tree phylo. *^2^D* + NO_3_^-^ | 7 | -160.98 | 337.86 | 2.41 | 0.15 | 0.48 |
|  | % ECM BA + ECM tree *^0^D* + NO_3_^-^ | 6 | -162.90 | 339.21 | 3.76 | 0.08 | 0.45 |
|  | % ECM BA + AM tree phylo. *^2^D* + NO_3_^-^ + C:N ratio + Forest floor mass | 8 | -160.62 | 339.72 | 4.27 | 0.06 | 0.48 |
|  | % ECM BA + Total (AM + ECM) tree *^0^D* + NO_3_^-^ | 6 | -161.00 | 340.61 | 5.16 | 0.04 | 0.43 |
|  | Null (intercept + random effect) | 3 | -182.13 | 370.64 | 35.19 | 0.00 | 0.03 |
| Soil ECMF genus *^2^D* | Total (AM + ECM) stem *^2^D* + pH + Soil respiration + Nitrification | 7 | -112.40 | 240.71 | 0.00 | 0.20 | 0.33 |
|  | % ECM BA + total (AM + ECM) stem *^2^D* + pH + Soil respiration + Nitrification | 8 | -112.16 | 242.80 | 2.09 | 0.07 | 0.33 |
|  | ECM IV + total (AM + ECM) stem *^2^D* + pH + Soil respiration + Nitrification | 8 | -112.25 | 242.98 | 2.27 | 0.06 | 0.33 |
|  | Total (AM + ECM) stem *^2^D* + Soil respiration | 5 | -116.31 | 243.60 | 2.90 | 0.05 | 0.27 |
|  | Total (AM + ECM) stem *^2^D* | 4 | -117.75 | 244.15 | 3.44 | 0.04 | 0.23 |
|  | Null (intercept + random effect) | 3 | -123.50 | 253.39 | 12.68 | 0.00 | 0.02 |
| Root ECMF genus ^0^D | % ECM BA + %N × Forest floor mass | 7 | -140.52 | 297.15 | 0.00 | 0.84 | 0.51 |
|  | % ECM BA + %N × Nitrification × Forest floor mass | 11 | -137.93 | 303.24 | 6.09 | 0.04 | 0.55 |
|  | Total (AM + ECM) tree phyl. *^2^D* + % ECM BA + %N × Nitrification × Forest floor mass | 12 | -137.81 | 306.12 | 8.97 | 0.01 | 0.55 |
|  | ECM tree *^0^D* + % ECM BA + %N × Nitrification × Forest floor mass | 12 | -137.81 | 306.13 | 8.97 | 0.01 | 0.55 |
|  | ECM BA *^2^D* + % ECM BA + %N × Nitrification × Forest floor mass | 12 | -137.83 | 306.16 | 9.01 | 0.01 | 0.55 |
|  | Null (intercept + random effect) | 3 | -160.35 | 327.12 | 29.96 | 0.00 | 0.10 |
| Root ECMF ^2^D | AM tree *^0^D* × % ECM BA | 6 | -107.87 | 229.29 | 0.00 | 0.21 | 0.26 |
|  | AM tree *^0^D* + % ECM BA | 5 | -109.48 | 230.05 | 0.76 | 0.15 | 0.22 |
|  | AM tree *^0^D* | 4 | -111.29 | 231.29 | 2.00 | 0.08 | 0.18 |
|  | AM tree *^0^D* + % ECM BA + N_min | 6 | -108.98 | 231.51 | 2.22 | 0.07 | 0.21 |
|  | AM tree *^0^D* + ECM BA | 5 | -110.28 | 231.65 | 2.35 | 0.07 | 0.20 |
|  | Null (intercept + random effect) | 3 | -114.93 | 236.27 | 6.98 | 0.01 | 0.07 |
|  |  |  |  |  |  |  |  |

^a^Models containing variables correlated with those in the top model, or those < 2 ΔAICc that are extensions of top model (more parameters) removed from model averaging

^b^+ = Without interaction terms; × = with interaction terms

^c^*K* = Number of parameters

^d^logLik = loglikelihood

^e^*W_i_* = AICc model weight

**Table S8** AICc table showing top-ranked models used for model-averaged predicted responses of soil and root ECMF functional diversity to tree abundance and diversity, as well as soil variables from the Adirondack Mountains, USA (2017)

| **Response** | **Model^ab^** | ***K*^c^** | **logLik^d^** | **AICc** | **ΔAICc** | ***W_i_*^e^** | **Cond. R^2^** |
| --- | --- | --- | --- | --- | --- | --- | --- |
| Soil ECMF funct. *^0^D* | ECM focal tree + Total (AM + ECM) phyl. *^0^D* + NO_3_^-^ + C:N ratio + pH + N mineralization | 9 | -60.69 | 142.60 | 0.00 | 0.19 | 0.40 |
|  | ECM focal tree + ECM tree phyl. *^0^D* + NO_3_^-^ + C:N ratio + pH + N mineralization | 9 | -61.03 | 143.27 | 0.67 | 0.14 | 0.39 |
|  | ECM focal tree + Total (AM + ECM) tree *^0^D* + NO_3_^-^ + C:N ratio + pH + N mineralization | 9 | -61.69 | 144.58 | 1.98 | 0.07 | 0.38 |
|  | Total (AM + ECM) tree PD *^0^D* + NO_3_^-^ + C:N ratio + pH + N mineralization | 8 | -63.06 | 144.65 | 2.04 | 0.07 | 0.35 |
|  | ECM focal tree × ECM tree PD *^0^D* + NO_3_^-^ + C:N ratio + pH + N mineralization | 10 | -60.36 | 144.71 | 2.11 | 0.07 | 0.41 |
|  | Null (intercept and random) | 3 | -77.34 | 161.06 | 18.46 | 0.00 | 0.00 |
| Soil ECMF funct. *^2^D* | ECM tree phyl. *^2^D* + Soil respiration + Nitrification + pH | 7 | -68.93 | 153.79 | 0.00 | 0.15 | 0.24 |
|  | Total (AM + ECM) stem *^2^D* + Soil respiration + Nitrification + pH | 7 | -69.34 | 154.62 | 0.83 | 0.10 | 0.23 |
|  | Soil respiration + Nitrification + pH | 6 | -70.78 | 154.99 | 1.20 | 0.08 | 0.20 |
|  | % ECM BA + ECM tree phyl. *^2^D* + Soil respiration + Nitrification + pH | 8 | -68.80 | 156.13 | 2.35 | 0.05 | 0.24 |
|  | Total (AM + ECM) stem *^2^D* + Soil respiration | 5 | -72.63 | 156.26 | 2.48 | 0.04 | 0.22 |
|  | Null (intercept + random) | 3 | -77.90 | 162.18 | 8.39 | 0.00 | 0.00 |
| Root ECMF funct. *^0^D* | ECM focal tree + ECM tree *^0^D* + Fine root biomass | 6 | -71.09 | 155.76 | 0.00 | 0.16 | 0.30 |
|  | ECM focal tree + total (AM + ECM) tree *^0^D* + Fine root biomass | 6 | -71.53 | 156.65 | 0.88 | 0.11 | 0.29 |
|  | ECM focal tree + nitrification + Fine root biomass + pH | 7 | -70.40 | 156.96 | 1.20 | 0.09 | 0.32 |
|  | ECM focal tree + Fine root biomass | 5 | -72.98 | 157.06 | 1.30 | 0.09 | 0.25 |
|  | ECM focal tree × total (AM + ECM) tree *^0^D* + Fine root biomass | 7 | -70.53 | 157.22 | 1.46 | 0.08 | 0.32 |
|  | Null (intercept and random) | 3 | -81.51 | 169.44 | 13.68 | 0.00 | 0.04 |
| Root ECMF funct. *^2^D* | ECM tree phyl. *^2^D* + %N + NO_3_^-^ + Soil respiration | 7 | -68.43 | 153.02 | 0.00 | 0.10 | 0.24 |
|  | Total (ECM + AM) tree phyl. *^0^D* + %N + NH_4_^+^ + NO_3_^-^ + Soil respiration | 8 | -67.89 | 154.61 | 1.60 | 0.04 | 0.25 |
|  | Total (ECM + AM) tree phyl. *^0^D* + Soil respiration | 5 | -71.90 | 154.92 | 1.90 | 0.04 | 0.15 |
|  | ECM BA + AM stem *^2^D* + Soil respiration | 6 | -71.10 | 155.78 | 2.76 | 0.02 | 0.17 |
|  | ECM BA + ECM tree phyl. *^2^D* + %N + NH_4_^+^ + NO_3_^-^ + Soil respiration | 9 | -67.13 | 155.86 | 2.85 | 0.02 | 0.27 |
|  | Null (intercept + random) | 3 | -76.58 | 159.59 | 6.57 | 0.00 | 0.00 |
|  |  |  |  |  |  |  |  |

^a^Models containing variables correlated with those in the top model, or those < 2 ΔAICc that are extensions of top model (more parameters) removed from model averaging

^b^+ = Without interaction terms; × = with interaction terms

^c^*K* = Number of parameters

^d^logLik = loglikelihood

^e^*W_i_* = AICc model weight

**Table S9** AICc table showing top-ranked models used for model-averaged predicted responses of relative abundance of medium distance-fringe (MD fringe) ECMF fungi to tree abundance and diversity, as well as soil variables from the Adirondack Mountains, USA (2017)

|  |  |  |  |  |  |  |
| --- | --- | --- | --- | --- | --- | --- |
| **Response** | **Model^a^** | ***K*^b^** | **logLik^c^** | **AICc** | **ΔAICc** | ***W_i_*^d^** |
| Soil MD fringe rel. abundance | AM tree *^0^D* + C:N ratio | 6 | 10.95 | -8.48 | 0.00 | 0.19 |
|  | C:N ratio + NH_4_^+^ | 6 | 10.71 | -8.00 | 0.48 | 0.15 |
|  | C:N ratio | 5 | 9.17 | -7.34 | 1.14 | 0.10 |
|  | C:N ratio + NH_4_^+^ + pH | 7 | 11.42 | -6.91 | 1.57 | 0.08 |
|  | AM tree *^0^D* + C:N + NH_4_^+^ + pH | 8 | 12.47 | -6.41 | 2.08 | 0.07 |
|  | Null (intercept + random) | 4 | 6.86 | -5.07 | 3.41 | 0.03 |
| Root MD fringe rel. abundance | ECM tree phyl. *^0^D* + C:N ratio | 6 | -0.51 | 14.60 | 0.00 | 0.30 |
|  | ECM tree phyl. *^0^D* + C:N ratio + pH | 7 | -0.44 | 17.04 | 2.44 | 0.09 |
|  | AM tree phyl. *^0^D* | 5 | -3.08 | 17.28 | 2.67 | 0.08 |
|  | ECM tree phyl. *^0^D* | 5 | -3.28 | 17.68 | 3.08 | 0.06 |
|  | C:N ratio | 5 | -3.56 | 18.24 | 3.64 | 0.05 |
|  | Null (intercept + random) | 4 | -4.90 | 18.54 | 3.93 | 0.04 |
|  |  |  |  |  |  |  |

^a^Models containing variables correlated with those in the top model, or those < 2 ΔAICc that are extensions of top model (more parameters) removed from model averaging

^b^*K* = Number of parameters

^c^logLik = loglikelihood

^d^*W_i_* = AICc model weight


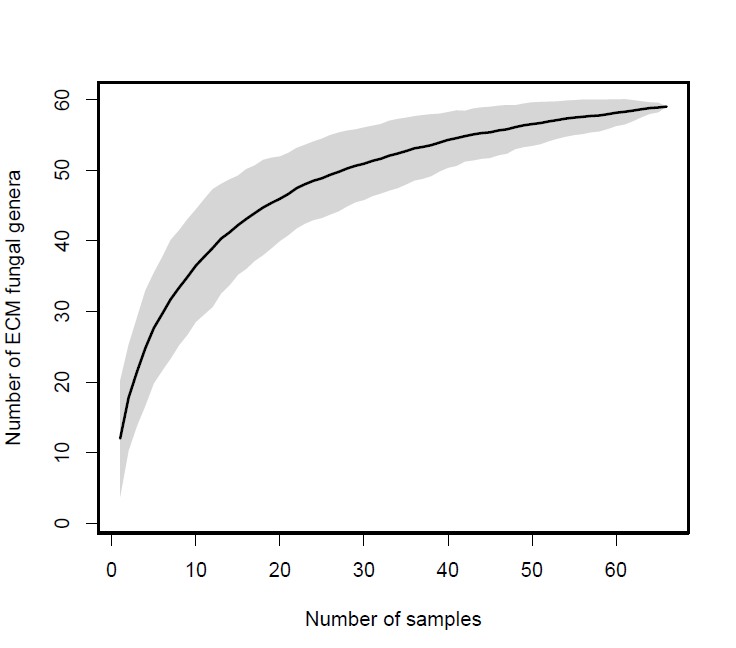


**Fig. S2** Accumulation curve of ECMF genera sequenced from soils from the Adirondack Mountains, USA (2017)


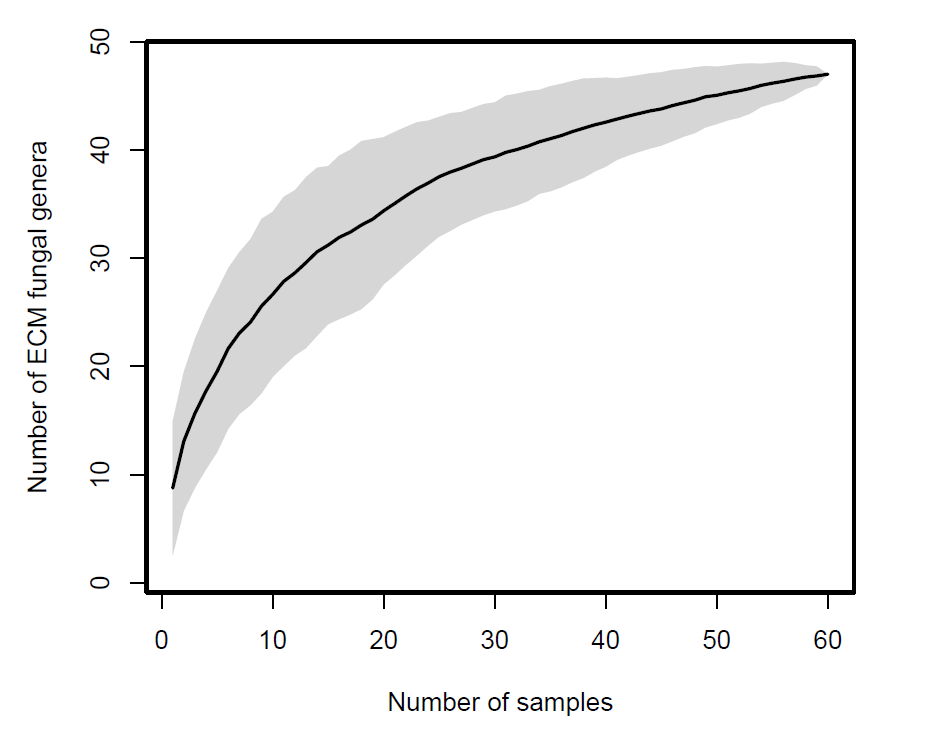


**Fig. S3** Accumulation curve of ECMF genera sequenced from roots from the Adirondack Mountains, USA (2017)


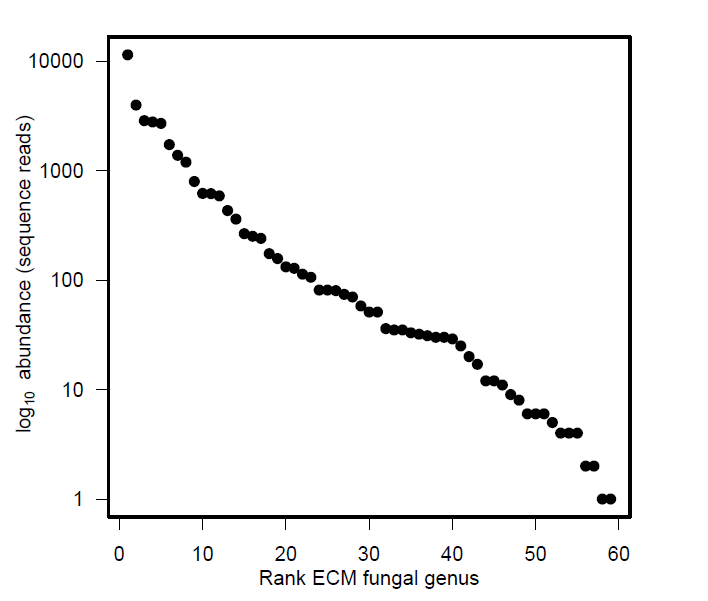


**Fig. S4** rank abundance curve for ECMF genera sequenced from soils from the Adirondack Mountains, USA (2017)


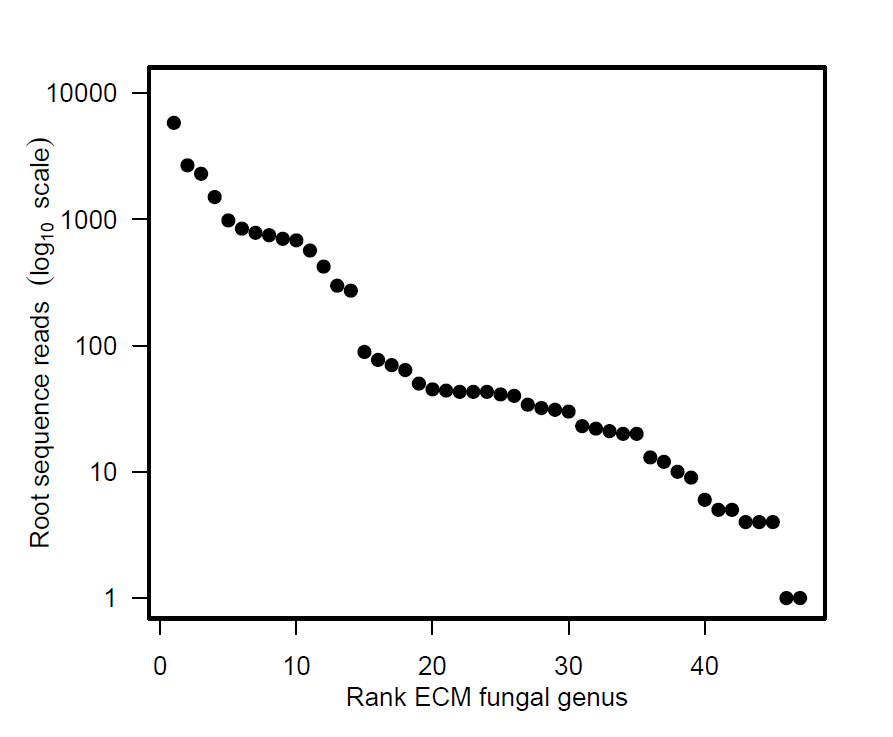


**Fig. S5** Rank abundance curve for ECMF genera sequenced from roots from the Adirondack Mountains, USA (2017)


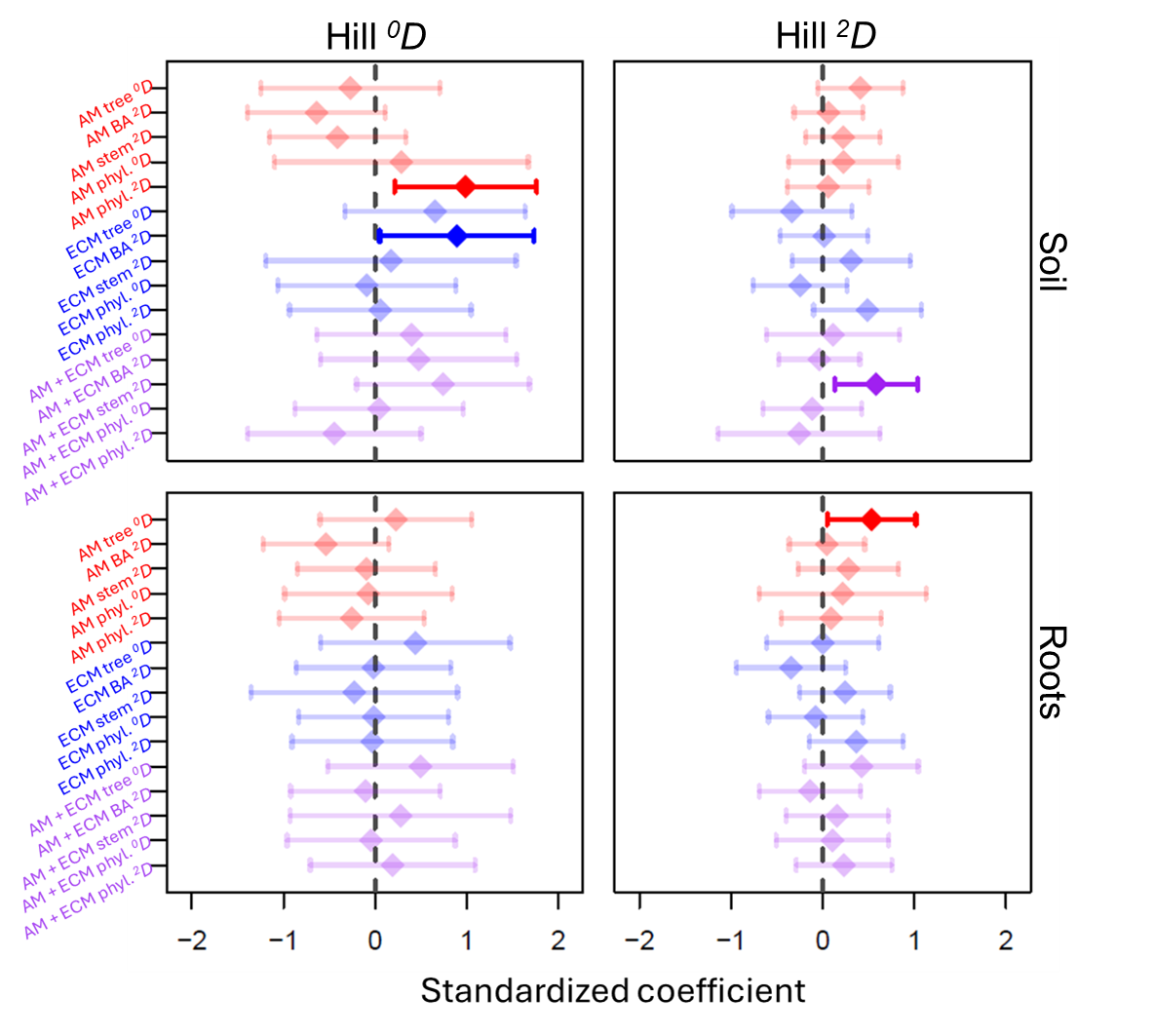


**Fig. S6** Model-averaged responses of ECMF taxonomic Hill *^0^D* (left) and Hill *^2^D* (right) from soil (top) and roots (bottom) in relation to arbuscular mycorrhizal (AM; top), ECM (middle), along with combined AM-ECM (bottom) tree phylogenetic and species Hill *^0^D* and *^2^D* diversity from the Adirondack Mountains, USA (2017). Soil variables included in top AICc-ranked models were also included for each response (Tables S7-S8). Bolded points indicate significant responses with 95% confidence intervals that do not intersect zero (dashed lines)


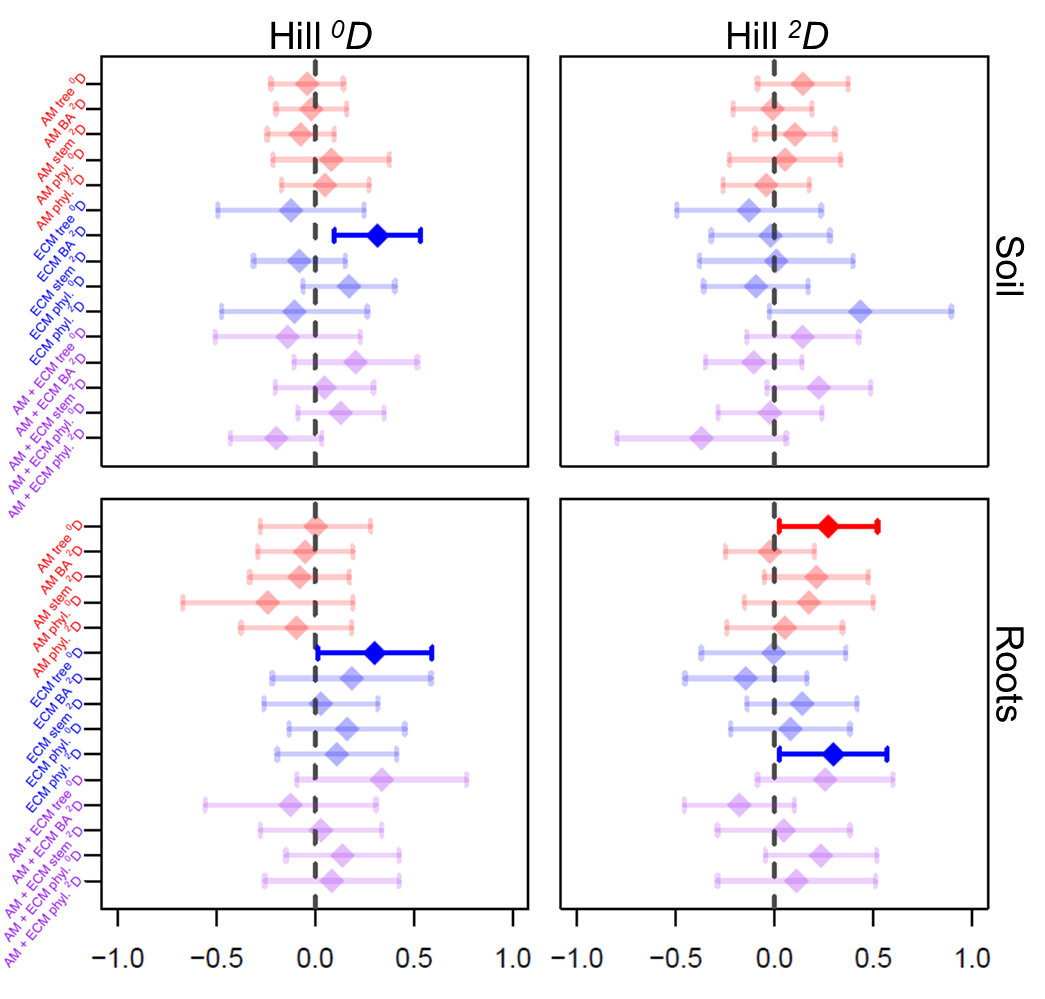


**Fig. S7** Model-averaged responses of ECMF functional Hill *^0^D* (left) and Hill *^2^D* (right) from soil (top) and roots (bottom) in relation to arbuscular mycorrhizal (AM; top), ECM (middle), along with combined AM-ECM (bottom) tree phylogenetic and species Hill *^0^D* and *^2^D* diversity from the Adirondack Mountains, USA (2017). Soil variables included in top AICc-ranked models were also included for each response (Tables S7-S8). Error bars represent 95% confidence intervals; bold colors represent significant variables with confidence intervals that do not intersect zero (dashed lines). Phyl = phylogenetic; BA = basal area

Table S10 Pearson correlations between arbuscular mycorrhizal (AM) trees and AM tree phylogenetic eigenvector from the Adirondack Mountains, USA (2017)

| Tree species | *r* | *p* |
| --- | --- | --- |
| *Acer pensylvanicum* | -0.373 | 0.001 |
| *Acer rubrum* | 0.328 | 0.005 |
| *Acer saccharum* | 0.272 | 0.022 |
| *Fraxinus americana* | -0.704 | <0.001 |
| *Hamamelis virginiana* | -0.108 | 0.371 |
| *Ulmus americana* | -0.304 | 0.010 |
|  |  |  |

Table S11 Pearson correlations between arbuscular mycorrhizal (AM) trees and soil pH from the Adirondack Mountains, USA (2017)

| Tree species | *r* | *p* |
| --- | --- | --- |
| *Acer pensylvanicum* | -0.261 | 0.028 |
| *Acer rubrum* | -0.286 | 0.016 |
| *Acer saccharum* | -0.373 | 0.021 |
| *Fraxinus americana* | 0.580 | <0.001 |
| *Hamamelis virginiana* | 0.269 | 0.023 |
| *Ulmus americana* | 0.279 | 0.018 |
|  |  |  |
